# Host diet shapes functionally differentiated gut microbiomes in sympatric speciation of blind mole rats in Upper Galilee, Israel

**DOI:** 10.3389/fmicb.2022.1062763

**Published:** 2022-11-15

**Authors:** Zhuoran Kuang, Fang Li, Qijiao Duan, Cuicui Tian, Eviatar Nevo, Kexin Li

**Affiliations:** ^1^State Key Laboratory of Grassland Agro-ecosystems, College of Ecology, Lanzhou University, Lanzhou, China; ^2^Department of Zoology, College of Life Sciences and Technology, Mudanjiang Normal University, Mudanjiang, China; ^3^Northwest Surveying and Planning Institute of National Forestry and Grassland Administration, Xi’an, China; ^4^Institute of Evolution, University of Haifa, Haifa, Israel

**Keywords:** metagenomics, subterranean mammals, host diet, microbiome community, sympatric speciation

## Abstract

The gut microbiome is important for host nutrient metabolism and ecological adaptation. However, how the gut microbiome is affected by host phylogeny, ecology and diet during sympatric speciation remain unclear. Here, we compare and contrast the gut microbiome of two sympatric blind mole rat species and correlate them with their corresponding host phylogeny, ecology soil metagenomes, and diet to determine how these factors may influence their gut microbiome. Our results indicate that within the host microbiome there is no significant difference in community composition, but the functions between the two sympatric species populations vary significantly. No significant correlations were found between the gut microbiome differentiation and their corresponding ecological soil metagenomes and host phylogeny. Functional enrichment analysis suggests that the host diets may account for the functional divergence of the gut microbiome. Our results will help us understand how the gut microbiome changes with corresponding ecological dietary factors in sympatric speciation of blind subterranean mole rats.

## Introduction

The gut microbiome plays a vital role in digestion, energy acquisition, detoxification, immune system development, behavior (Zhang et al., 2022), and driving host niche differentiation (Greene et al., 2020). Multiple factors may affect the gut microbiome community and function (McFall-Ngai et al., 2013), including environment (Spor et al., 2011), host diet (Maurice et al., 2015; Smith et al., 2015), host phylogeny (Maurice et al., 2015; Smith et al., 2015), and evolutionary history (Groussin et al., 2017; Youngblut et al., 2019). The composition of the gut microbiome is reported to be influenced by both host factors (e.g., host genetics and evolutionary history; Benson et al., 2010; Blekhman et al., 2015) and environmental factors (e.g., diet, geography; Frese et al., 2015). Several studies have estimated the effects of different factors on the gut microbiome composition and have obtained different conclusions. Some have found that the gut microbiome composition can influence host evolution and mirror the host’s phylogeny (Phillips et al., 2012). Different species always harbor distinct gut microbiomes; if transplantation of gut microbes is performed between species, it results in decreased fitness, e.g., a reduction in survival rates (Brooks et al., 2016). Geographic proximity does not indicate similar gut microbiomes between different host species (Ochman et al., 2010); even with sympatric distributions and large dietary overlaps, the gut microbiome exhibits strong host specificity (Yildirim et al., 2010). Some studies suggest that host genetics has little effect on the gut microbiome (Rothschild et al., 2018), and that ecological factors play a more dominant role (Rothschild et al., 2018; Liu et al., 2021). Studies of the gut microbiome from sympatric Madagascar lemurs fed on different diets demonstrated that the gut microbiome could recover host phylogeny, showed significantly differentiated clusters (Perofsky et al., 2019; Greene et al., 2020), and that dietary specializations enabled sympatric species to avoid competition and coexist (Schoener, 1974). The gut microbiome can also contribute to speciation in several ways. For example, they can affect the cuticular hydrocarbons (CHCs) on the host’s body surface to influence individual recognition (Sharon et al., 2010) and mating (Vernier et al., 2020). Though there is evidence to suggest that the gut microbiome may affect speciation, the causal relationship between the two remains controversial. Did the gut microbiome differentiation precede speciation and then promote species formation, or did host speciation shape the gut microbiome? We hypothesize that new ecological niches, including new diets, allow new species to survive, and that the gut microbiome slowly changes and adapts to these changes. We hypothesize that the function of gut microbiome is initially affected because of different host diets, followed by the species genetic and phenotypic composition (Uritskiy et al., 2019).

The blind mole rat, *Spalax*, belonging to the Spalacidae family, is an herbivorous subterranean mammalian rodent that lives most of its life underground (Nevo, 1961). Five species of the *Spalax ehrenbergi* Superspecies evolved in Israel four peripatric chromosomal species (Nevo et al., 1991; Nevo, 2001), and the fifth speciated sympatrically, genically but non-chromosomally (see all five species analyzed genomically in Li et al. (2020b). Two *Spalax galili* populations, *S. galili* basalt and *S. galili* chalk, (both 2*n* = 52) live in abutting but contrasting geologies and soils from “Evolution Plateau” eastern Upper Galilee, Israel. The slightly alkaline rendzina soil weathered from Senonian chalk at approximately 99.6 Mya, and the acid basalt soil, weathered from Pleistocene basalt, was generated from a volcanic eruption of about 1 Mya. The chalk is much drier and barren, compared to the basalt which has a clay consistency, and is wetter and muddier. There are 113 species of plants in total from the two soils, but only 28% are common in the different soil types (Hadid et al., 2013). The food resource diversity is much higher in basalt than in chalk (Lövy et al., 2015, 2017). There are more geophytes in basalt, whereas the bushlets of *Sarcopoterium spinosum* conquered the majority of the chalk soil (Lövy et al., 2017; Jiao et al., 2021). The geophytes are more nutritious for the blind mole rats (Mohammad and Alseekh, 2013), whereas those from the chalk feeding on primarily on roots may experience limited and low-quality food supply. These differentiated ecological variables may lead to divergent metabolism, population density (Hadid et al., 2013), and genetic clusters (Li K. et al., 2015; Li et al., 2016). *S. galili* chalk is the ancestor species ich later migrated into the rich basalt soil when it cooled down, and sympatrically speciated there to derive into *S. galili* basalt (Li K. et al., 2015; Lövy et al., 2015, 2017; Li et al., 2016). Although environmental, host diet, and phylogeny diverged during this speciation event, whether their gut microbiome diverged remains unknown. The clearly different host diets, contrasting environmental edaphic differences and separated host phylogeny of *Spalax*, supplied us with an ideal model to study this question.

In this study, we hypothesized that the host diet plays an important role in differentiating gut microbiomes, facilitating host population adaptation to the local environment, and aiding in further speciation. Here we compared the gut microbiome from the sympatrically speciated blind mole rat populations, measured the community composition and functional differences, and correlated it with the soil metagenomics and host phylogeny to test which factor contributed most to the gut microbiome divergence. These results will help us understand how the gut microbiome was influenced and whether they were involved in potential speciation (Bahrndorff et al., 2016).

## Materials and methods

### Single nucleotide polymorphism calling, filtering and phylogeny of *Nannospalax galili*

We downloaded the reference genome of *Nannospalax galili* (GCF_000622305.1) and pair-end (PE) reads from NCBI at https://www.ncbi.nlm.nih.gov/. PE reads were filtered by removing low-quality reads and adapters with fastp v0.20.1 (Chen S. et al., 2018). The clean reads were mapped to the reference genome with BWA-MEM v0.7.17-r118 15 (Li and Durbin, 2009), sorted and indexed with SAMtools v1.11 (Li H. et al., 2009). The duplicates were marked and variants were detected using GATK v4.0 16 (McKenna et al., 2010). SNPs were extracted and performed hard filtering by GATK with default parameters. Then SNPs were further filtered using VCFtools v0.1.16 (Danecek et al., 2011) with parameters: --maf 0.05. --minDP 5. --maxDP 50. --minGQ 20. --hwe 0.01 and --max-missing 0.9. A genetic distance matrix was generated by PLINK (Purcell et al., 2007). We next used FastME 2.0 (Lefort et al., 2015) to construct a phylogenetic tree with default parameters.

### Sampling, DNA isolation, and sequencing

In total, 12 gut samples were collected for chalk (6 samples) and its abutting derivative basalt (6 samples) from “Evolution Plateau” (EP): in the eastern Upper Galilee, Israel, in January 2016 and were stored in liquid nitrogen immediately for DNA extraction. DNA was isolated using QIAmp DNA stool mini kit (Qiagen, United States). Qualified DNA was sonicated to about 350 bp fragments, after purification, end-repair, A-tailing, and adaptor ligation were carried out and followed by PCR amplification. After quality control, libraries of each sample were sequenced with 150 bp paired-end reads. We generated ~50 million 150 bp PE raw reads from the shotgun metagenomic sequencing for each sample. Paired-end raw sequencing reads were first trimmed by SOAPnuke (Chen Y. et al., 2018) to remove adapters and low-quality bases, and the host reads (*S. galili*) were removed by SOAPaligner (Li R. et al., 2009). We also collected 12 soil samples for shotgun metagenomic sequencing at the same location (Mukherjee et al., 2022), and the data of soil were processed as described below.

### Taxonomy annotation

First, clean reads were uploaded into the MG-RAST (Meyer et al., 2019) server, and bacteria were found to be the most dominant kingdom (over 99.5%). Kraken2 v2.1.2 (Wood et al., 2019) was used for taxonomic profiling, and the relative abundance was estimated by Bracken v2.5 (Lu et al., 2017). Meanwhile, alpha and beta diversity were calculated using R packages “vegan”.^1^ Species with significant abundance differences between basalt and chalk were identified using LEfSe (linear discriminant analysis effect size; Segata et al., 2011) on the Galaxy website (v1.0).^2^ We generated a Bray-Curtis distance matrix based on the microbiome composition of the gut using R packages “vegan,” and the microbiota dendrogram was constructed based on the distance matrix by R packages “ape.” Mantel-test was performed to compare the relationship between the host genetic distance matrix and Bray-Curtis distance matrix based on the gut microbiome. Procrustes analysis and species accumulation curves were performed on the Tutools platform.^3^ Statistical analysis was conducted with R.

### Metagenomic assembly and binning

Contigs were assembled by MEGAHIT v1.1.3 (Li D. et al., 2015) with default parameters. After assembly, assembled contigs were binned, refined, and reassembled by metaWRAP v1.3.2 (Uritskiy et al., 2018). The completeness and contamination of each bin were evaluated by CheckM v1.0.12 (Parks et al., 2015). All bins were aggregated and then dereplicated using dRep v3.2.2 (Olm et al., 2017; parameters: -comp 50 -con 10 -pa 0.90 -sa 0.95 -nc 0.30 -cm larger). The taxonomy of bins was assigned using GTDB-tk v1.7.0 (Chaumeil et al., 2020) and these results were visualized in iTOL v6 (Letunic and Bork, 2007) as a phylogenetic tree. The relative abundance of bins was estimated by CoverM v0.6.1.^4^ Bray-Curtis distance matrix and PCoA were generated by R packages “vegan,” and the heatmap was plotted by https://www.bioinformatics.com.cn.

### Construction of non-redundant gene catalog and function annotation

The coding sequences (CDS, > 100 bp) were predicted by Prodigal v2.6.3 (Hyatt et al., 2010), and we used CD-HIT v4.8.1 (Fu et al., 2012; parameters: -c 0.95 -d 0 -aL 0.9 -uL 0.05 -aS 0.9) to remove redundancy. The non-redundant gene catalog was searched against the Kofam database by KofamKOALA v1.3.0 (Aramaki et al., 2020). Because KEGG Orthology and KEGG pathway have a many-to-many relationship, we used ReporterScore (Bäckhed et al., 2015) and biostack-suits^5^ to analyze all KOs and used the overall trend to reflect the change in the pathway. Carbohydrate-active enzymes (CAZyomes) were identified by dbCAN2 (Zhang et al., 2018). Clusters of Orthologous Groups of proteins (COG) and Gene Orthology (GO) were determined by eggNOG-mapper v2.1.6 (Huerta-Cepas et al., 2017). Meanwhile, alpha diversity and beta diversity were also calculated by R packages “vegan.” Significantly different GO terms and CAZyomes between basalt and chalk were identified by STAMP v2.1.3 (Parks et al., 2014). Statistical analysis was conducted with R and significant outliers were removed. The relative gene abundance was calculated as follows (Qin et al., 2012; Liu et al., 2021).

Step 1: Clean reads were aligned to the non-redundant gene catalog using BWA (Vasimuddin et al., 2019), and genes with a number of mapped reads less than two were removed; the copy number of each gene was: b_i_ = x_i_/L_i_;

Step 2: Calculation of the relative abundance of gene i: a_i_ = b_i_/∑b_i;_

a_i_: the relative abundance of gene i.

b_i_: the copy number of gene i from sample N.

L_i_: the length of gene i.

x_i_: the number of mapped reads.

The calculations mentioned above were performed with custom Python scripts.

### Single nucleotide polymorphisms analysis of metagenomics

First, we used kraken2 v2.1.2 to identify the most abundant species and significantly different species were identified by LEfSe; second, we downloaded their genomes from NCBI as references, but the reference genome of *Akkermansia muciniphila* we used was generated in the present study. SNPs were detected the same as described above with the following parameters: “--maf 0.05. --hwe 0.01 and --max-missing 0.9.” Principal component analysis (PCA) was accomplished by PLINK v1.90b6.21 (Purcell et al., 2007). Neighbor-joining (NJ) tree was conducted by TreeBeST v1.9.2 (Vilella et al., 2009). Demographic history was analyzed by SMC++ (Terhorst et al., 2017). *F*_ST_ and nucleotide diversity (π) were calculated by VCFtools in 1 kb sliding windows.

Because the abundance of *Flavonifractor plautii* is also high in the host dwelling soil, so we compared both the gut and soil microbiome to investigate the differentiation of this species. Furthermore, admixture v1.3.0 (Alexander and Lange, 2011) was used to perform structural analysis with the number of clusters (K) ranging from 2 to 4, respectively. A genetic distance matrix was generated by PLINK, and visualization of the genetic network was finished by R packages (“netview,” “network,” “graph,” “sna,” “visNetwork,” “three,” and “networkD3”) with k = 7 (Steinig et al., 2016).

### Sampling, protein extraction, and label-free analysis

We collected eight liver samples of the host *Spalax*, including four individuals from basalt and four from chalk, for proteome comparison. Tissues were frozen in liquid nitrogen immediately and stored at −80°C. The samples were ground first, then add 0.4 ml protein cracking liquid, containing 100 mM Tris–HCl (pH 8.0), 10 mM DTT, 8 M urea, and 1X protease-inhibitor. The mixture was incubated on ice for 30 min and then centrifuged at 10,000 g and 4°C for 30 min. The supernatant was collected, and protein concentration was determined with a BCA protein assay kit. Protein quality was identified with SDS-PAGE.

Protein samples were diluted with NH_4_HCO_3_ (200 mM), and incubated with 10 mM DDT for 1 h at 56°C, after cooling, add 55 mM iodoacetamide (IAA) for 40 min in the dark to alkylate samples, then digested with 5 μg trypsin for 14-16 h at 37°C. Digested peptides were concentrated to about 1 mg/ml, then separated with chromatography using Eksigent 425 (AB SCIEX). Separated peptides were performed mass spectrometric analysis with Q-Exactive (Thermo Scientific).

Raw files were extracted by MaxQuant (Cox and Mann, 2008). MS data were searched against Uniprot- Heterocephalus glaber database with parameters: max missed cleavages were 2; Carbamidomethylation (C) were set as fixed modifications, and oxidization (M) were set as variable modifications; Peptide Mass Tolerance was ±15 ppm; fragment mass tolerance was 20 mmu; peptide length was >4. The cutoff of the global false discovery rate (FDR) for peptide and protein identification was set to 0.01. Then screening proteins based on the threshold with a minimum fold change of 1.5 (*p* < 0.05). Statistical analysis was conducted by Student *t* -test with Microsoft Excel. For proteins with missing values, data for that protein were kept if its lowest value in one group was higher than its highest value in another group. Finally, Gene ontology (GO) enrichment, Kyoto Encyclopedia of Genes and Genome (KEGG) pathway enrichment analysis were performed to characterize the properties of the proteins identified using STRING (Szklarczyk et al., 2015), these results were visualized by https://www.bioinformatics.com.cn.

## Results

### Metagenomic assembly and binning

**Figure 1:**
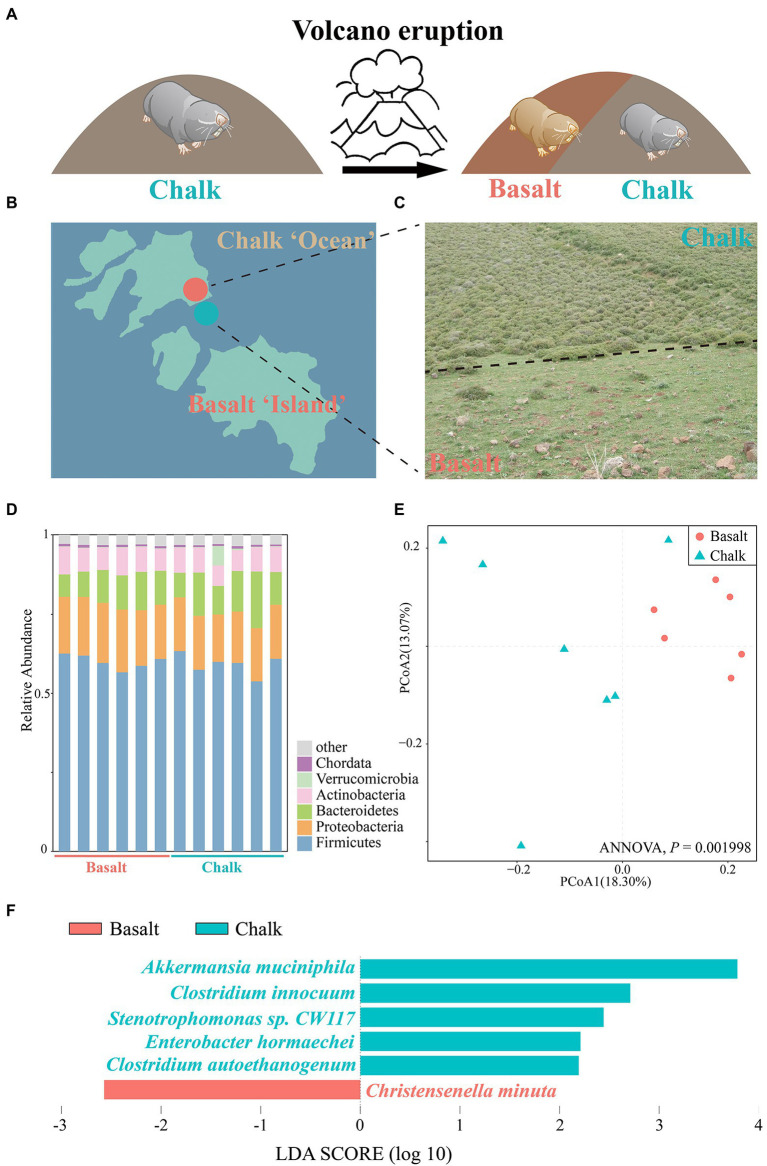
Abutting but different environments and the gut microbiome composition. **(A)** Volcanic eruptions have led to the formation of new habitats and new populations of Basalt. **(B)** Geological map including the Senonian chalk soil and the abutting derivative Plio-Pleistocence basalt soil, which is like reddish basaltic islands in pale chalk ocean. **(C)** Habitat-specific photographs and the contrasting plants with only 28% of the same in the abutting different soils. **(D)** Barplot of Bacteria composition, which showed that the most abundant phyla are Firmicutes and Proteobacteria. **(E)** Principal coordinates analysis (PCoA) based on the relative abundance of MAGs showed samples from basalt clustered together and samples from chalk were in one cluster. **(F)** Species with significant differences in chalk and basalt, identified by LEfSe.

A total of ~95 GB of raw reads (Supplementary Table 1) from the shotgun metagenomic sequencing was generated, and ~ 80 GB of clean reads (Supplementary Table 1) were retained for six basalt and six chalk gut samples after trimming the low-quality bases. We obtained an average of 570,905 contigs (>200 bp) for each metagenomic assembly for downstream analysis (Supplementary Table 2). In total, 339 bins (>50% completeness, <10% contamination) were obtained (Supplementary Figures 1, 2), with 139 high-quality bins as defined by Bowers et al. (2017; >90 complete, <5 contamination). All bins can be annotated at the family level (Supplementary Table 3), with the majority of bins (614 bins, 90.4%) also annotated at the genus level (Supplementary Table 3), indicating potential new species in the gut. One bin (Chalk3.37) can be annotated to the species level (*Akkermansia muciniphia*). PCoA based on the relative abundance of all bins showed a clear separation between the chalk and basalt populations (Figure 1E; ANNOVA, *p* = 0.001998). The heat map also showed similar results (Supplementary Figure 2), indicating that chalk and basalt differ at the strain level.

### Gut microbiome community composition in basalt and chalk

We checked the sample size saturation and found that as the sample number increased to nine the species accumulation curve (Supplementary Figure 3) began to plateau, indicating that our 12 samples were sufficient to cover most species. We characterized the overall microbiome composition variation by PCoA based on Bray-Curtis distance and found there was no significant difference between the chalk and basalt (Supplementary Figure 4; ANNOVA, *p* = 0.314). Additionally, chalk and basalt did not differ significantly in alpha diversity (Supplementary Figure 5). We found *Akkermansia muciniphila*, *Clostridium innocuum*, *Stenotrophomonas* sp. *CW117*, *Enterobacter hormaechei*, *Clostridium autoethanogenum* were overrepresented in chalk, and *Christensenella minuta* was enriched in basalt (Figure 1F; LDA score (log10) > 1.5).

**Figure 2:**
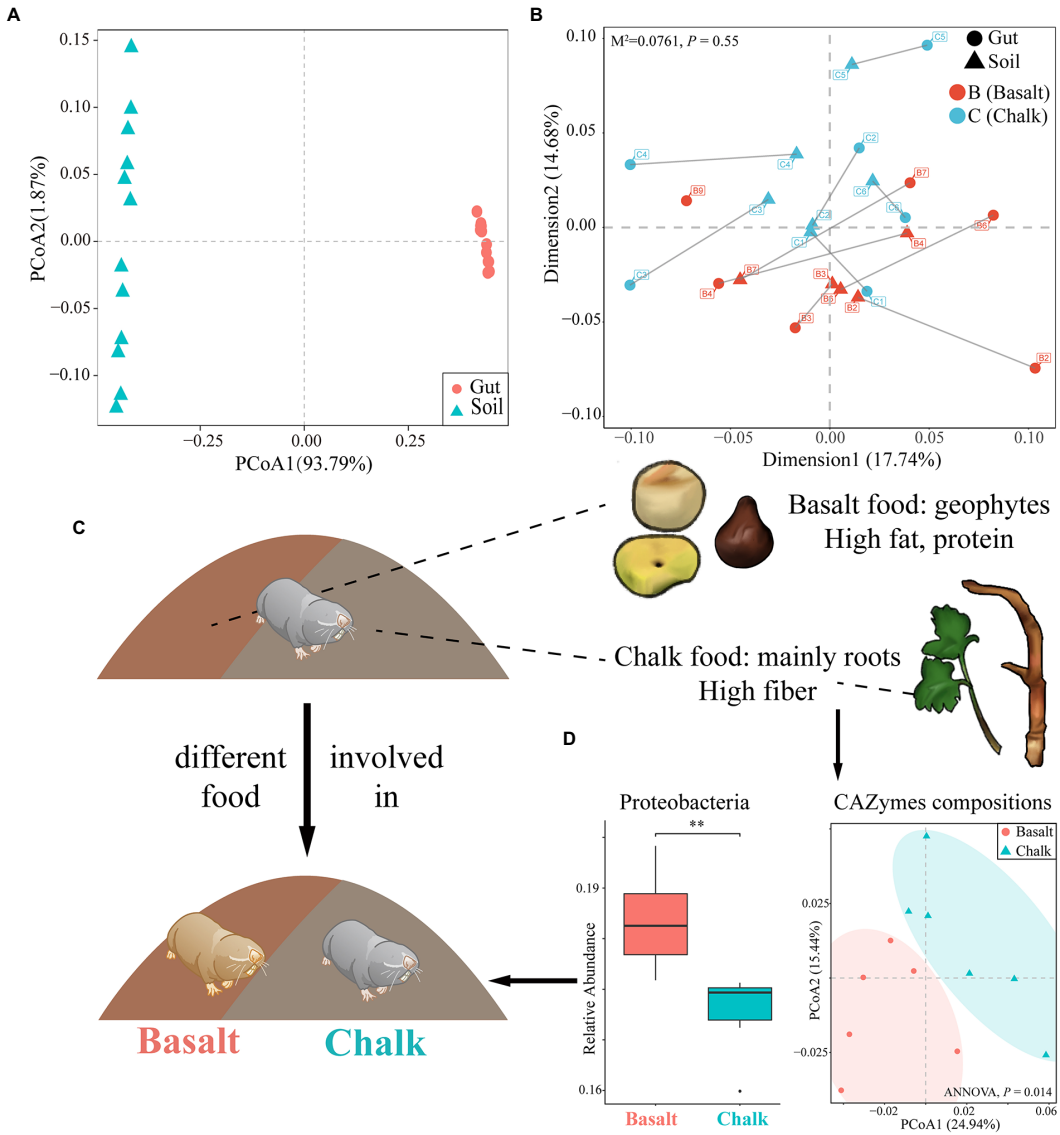
The effect of host diet, phylogeny and soil metagenomics on the gut microbiome. **(A)** Principal component analysis showed that the community composition of gut and soil differ significantly. **(B)** Procuster analysis showed no correlation in abundance between gut microbiome composition and soil microbiome composition. **(C)** The emergence of new habitats was followed by new foods, which may have been involved in host divergence. Basalt’s diet was primarily geophytes, rich in fat and protein; chalk’s diet was mainly roots, rich in fiber. This also shaped their gut microbiome. **(D)** The basalt was richer in Proteobacteria, and the CAZymes compositions of the two were significantly different, which are characteristics of their adaptation to different diets. **p<0.01.

The most dominant kingdom in both populations gut microbial communities were bacteria (over 99.5%), followed by archaea and eukaryota. For the bacterial community, 57 phyla, 99 classes, 214 orders, 469 families, 1721 genera, and 6,401 species were identified from 12 gut samples. We found 2 phyla, 8 classes, 16 orders, 24 families, 109 genera, and 313 species were significantly different between basalt and chalk (Welch’s *t*-test, *p* < 0.05) populations. In both populations, the most abundant phyla were Firmicutes that accounted for 59.6% of abundance, followed by Proteobacteria (17.4%), Bacteroidetes (10.7%), ActinobacteriaI (7.61%) and others (4.57%, Figure 1D). Paenibacillus (6.21%), Lachnoclostridium (5.83%), Flavonifractor (4.24%), Oscillibacter (3.99%) and Blautia (3.79%) were the most abundant genera. It is worth noting that Chalk-3 contained considerable *Akkermansia muciniphila* which belonged to the Verrucomicrobia phylum, Akkermansia genus (6.09%, Figure 1D). Moreover, we performed Welch’s *t*-test and found significant difference in relative phylum abundance of Proteobacteria (Figure 2D; *p* = 6.32e-3) and Candidatus Micrarchaeota (*p* = 0.013). At the genus level, Christensenella, Acidovorax, Bordetella, Bibersteinia and Faecalitalea were enriched in basalt; While in chalk, Providencia, Acidithiobacillus, Gimesia, Gemella and Tardiphage were enriched. The ratio of Firmicutes to Bacteroidetes (F/B ratio) was higher in the basalt compared to chalk (Supplementary Figure 6). We also compared the microbial diversity between the soil and gut and found significant differences in community composition (Figure 2A) and significantly higher Shannon diversity in the soil microbiome (Supplementary Figure 7). Meanwhile, Procruster analysis (Figure 2B; M^2^ = 0.0761, *p* = 0.55) and mantel test (r = − 0.01518, *p* = 0.5317) showed there was no correlation between the gut and soil microbiomes.

### Non-redundant gene catalog and functional annotation

**Figure 3:**
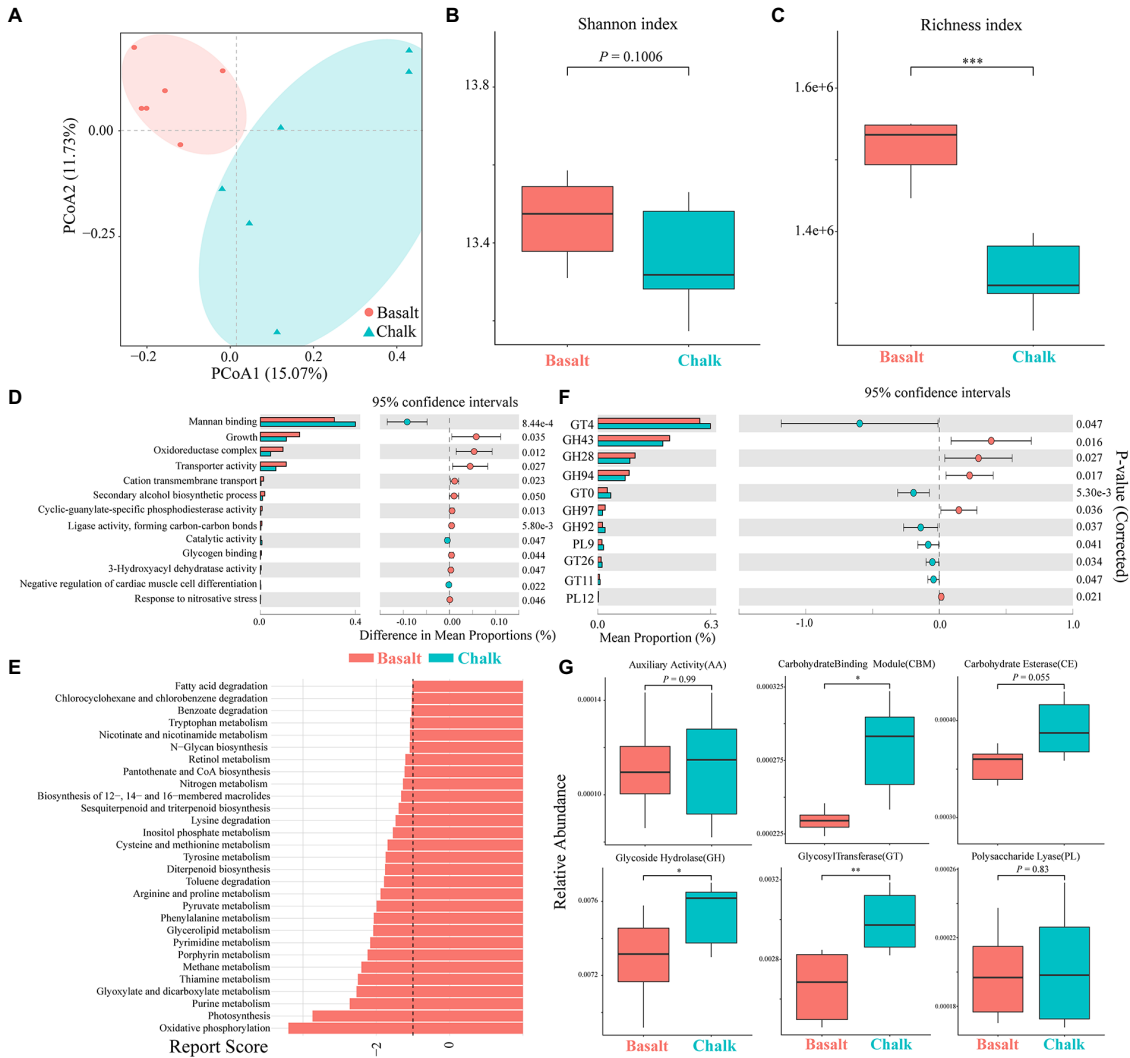
Functional compositions of gut microbiomes of basalt and chalk. **(A)** Principal component analysis shows that the function compositions were clearly separated. **(B)** Basalt has a higher shannon index based on non-redundant gene sets. **(C)** Basalt has a higher richness index based on non-redundant gene sets. **(D)** GO terms with significant differences between basalt and chalk. **(E)** KEGG pathways with significant differences between basalt and chalk. **(F)** Carbohydrate active enzymes (CAZyomes) with significant differences between basalt and chalk. **(G)** Comparison of abundances of different CAZYomes families between basalt and chalk. *p<0.05, **p<0.01, and ***p<0.001.

We plotted the cumulative curves for KEGG, GO and CaZy and all curves began to plateau, indicating that our 12 samples were sufficient to reveal the function of both communities (Supplementary Figure 8). Principal coordinate analysis (PCoA) of the non-redundant gene catalog based on Bray-Curtis distance showed that the functional composition of basalt and chalk was different (Figure 3A; ANNOVA, *p* = 0.00995). The Shannon index of basalt was higher than that of chalk (Figure 3B) but was not significantly different. The richness index, however, was significantly higher in chalk (Figure 3C; *p* = 0.0039). For GO terms, the non-redundant genes of basalt were enriched in growth, oxidoreductase complex, transporter activity, cation transmembrane, etc. (Figure 3D), and enriched in mannan-binding, catalytic activity, and negative regulation of cardiac muscle cell differentiation in chalk. The significantly enriched KEGG pathways (Figure 3E) in basalt included: Oxidative phosphorylation, Purine metabolism, Glyoxylate and dicarboxylate metabolism, Methane metabolism, etc. CAZymes (carbohydrate-active enzymes) compositions were significantly different (ANNOVA, *p* = 0.014; Figure 2D) between basalt and chalk. The diversity (Supplementary Figure 9) and abundance (Figure 3F) of CAZymes were higher in chalk, which contains more (Figure 3G) Carbohydrate-binding module (CBM), carbohydrate esterase (CE), glycoside hydrolase (GH) and glycosyl transferase (GT). GH43, GH28, GH94, GH97 and PL12 were enriched in basalt, while GT4, GT10, GH92, PL9, GT26 and GT11 were enriched in chalk. For COG (Cluster of Orthologous Groups database) annotations (Supplementary Figure 10), the metabolism category was dominant in both chalk and basalt; cellular processes and signaling were the second most dominant. Furthermore, Replication, recombination and repair (COG L) was the most abundant COG type, followed by Carbohydrate transport and metabolism (COG G) and Amino acid transport and metabolism (COG E; Supplementary Figure 10). Recombination and repair (COG L) were enriched in basalt (Supplementary Figure 11C). Transcription (COG K), cell wall/membrane/envelope biogenesis (COG M) and energy production and conversion (COG C) were enriched in chalk (Supplementary Figures 11A,B,D).

### Genetic divergence between the two microsites

**Figure 4:**
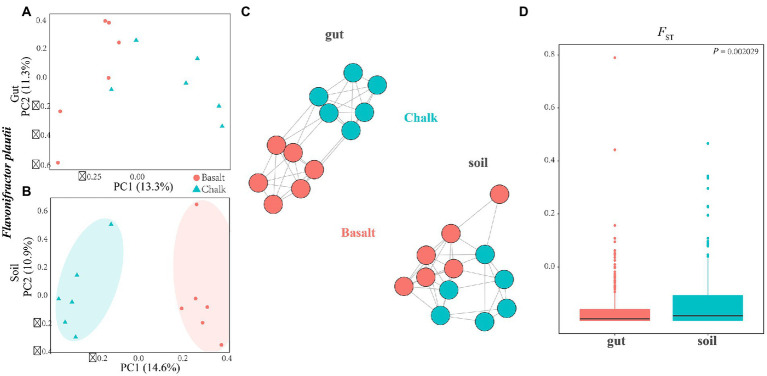
Sympatric divergence of the single bacteria of *Flavonifractor plautii* in gut and soil*.* **(A)** Principal component analysis shows the gut microbiome from basalt clustered together and separated with samples from chalk were in one cluster. **(B)** In soil, principal component analysis shows samples from basalt clustered together and samples from chalk were in one cluster. **(C)** Genetic network analysis of this species in the environmental soils and gut. **(D)** *F*_ST_ for *Flavonifractor plautii* was significantly higher in soils than that in gut.

The species identified by LEfSe were similar between the chalk and the basalt (Supplementary Figures 26–31) populations; however, many species showed separate genetic clusters between basalt and chalk measured by PCA (Figure 4A; Supplementary Figures 12A–31A), phylogenetic tree (Supplementary Figures 12A, 13B–31B) and genetic networking (Figure 4C) based on SNPs. Demographic analysis revealed that microbiome differed between chalk and basalt, but the differentiation was not high (Supplementary Figures 12B, 13C–31C). Interestingly, the differentiation time of *Akkermansia muciniphila* between the chalk and the basalt populations is estimated to be approximately 6,000 to 7,000 years. These changes have occurred more recent than the genetic differentiation of the two host species which is estimated to be approximately 228,000 years 33 by SMC++ analysis (Supplementary Figure 26C). Other microbial species also have a shorter differentiation time (Supplementary Figures 26–31). *F*_ST_ and nucleotide diversity (π) were also calculated for these species based on SNPs (Supplementary Table 4).

Furthermore, we explored the differentiation of the same bacterial species of *Flavonifractor plautii* between the chalk and basalt gut microbial composition and the two types of soils using PCA (Figures 4A,B) and genetic networking (Figure 4C). We revealed that it was separated into two clear-cut clusters in the soil but not in the gut (Figures 4C,D). *F*_ST_ was also calculated for both the gut and soil, and it was significantly higher in the soil than in the gut microbiome (Figure 4D). Compared to the genetic differentiation of the hosts between the chalk and basalt, the differentiation of the gut microbiome is later and smaller, indicating that the gut microbiome was not a major factor influencing host divergence. When the individuals were separated into two groups (K = 2), the basalt population is completely separated from the chalk population (Supplementary Figures 32, 33).

### Host divergence and the correlation with its gut microbiome

**Figure 5:**
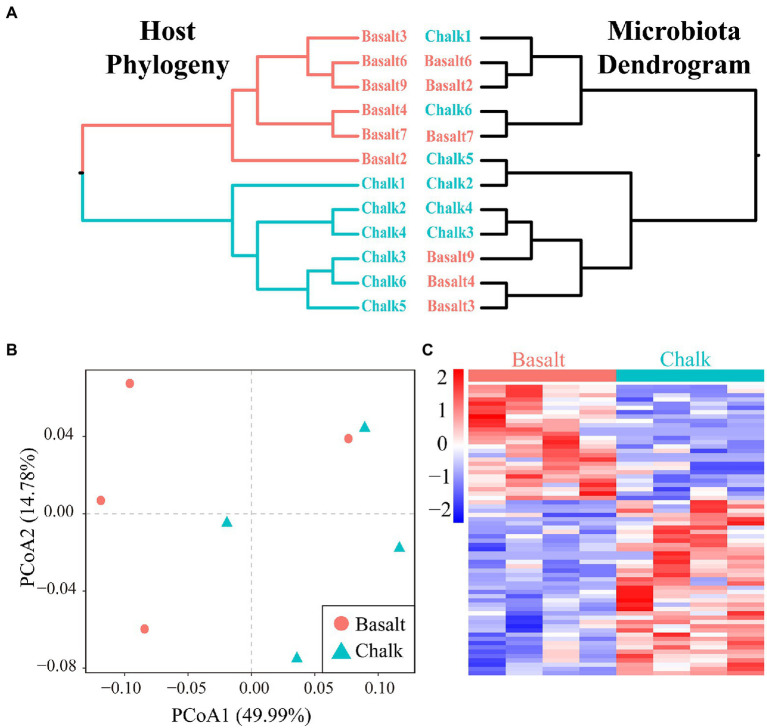
Host divergence and the correlation with its gut microbiome. **(A)** Host phylogeny and microbiota dendrogram were not mirroring each other. **(B)** Principal component analysis showed that the protein composition of basalt and chalk did not differ significantly. **(C)** Abundance clustering Heatmap of 127 differential proteins.

We performed a mantel test and found there was no correlation (*r* = 8.078e-3, *p* = 0.4528) between the host genetic matrix and the Bray-Curtis distance matrix based on the gut microbiome. The microbiome dendrogram did not reflect the host phylogeny (Figure 5A). PCoA based on protein abundance showed little difference in protein composition between the hosts from basalt and chalk (Figure 5B). However, we did identify 127 significant differential proteins (Figure 5C; *p* < 0.05 Student *t*-test) that were significantly enriched in metabolic pathways such as endocytosis, biosynthesis of amino acids, endocrine and other factor − regulated calcium reabsorption, arginine and proline metabolism, vasopressin−regulated water reabsorption, and pyruvate metabolism (Supplementary Figure 34). GO enrichment of Biological Process and Molecular Function also showed similar results (Supplementary Figure 34): differential proteins were mainly involved in the synthesis of proteins (e.g., Organonitrogen compound metabolic process, cellular metabolic process, translation, Peptide biosynthetic process), they may be secreting proteins (e.g., Regulation of multivesicular body size and Retrograde transport, endosome to the plasma membrane), and were followed by the synthesis and decomposition of various biological substances (e.g., Amide biosynthetic process, Negative regulation of cholesterol biosynthetic process and Fumarate metabolic process). Enrichment of GO Cellular Component (Supplementary Figure 35) also indicated that these proteins may be secretory proteins or have transport functions (e.g., Cytoplasmic vesicle, Late endosome, and Clathrin-coated vesicle membrane).

## Discussion

The gut microbiome is essential for host digestion (Miller et al., 2020) and health (de Vos et al., 2022) and may facilitate the hosts ability to adapt to the local environment (Greene et al., 2020). In this study, we compared the gut microbiome from the sympatrically speciated species *S. galili* chalk and *S. galili* basalt (Li K. et al., 2015). The ancestral chalk species is from Senonian, while the basalt species is from a volcanic eruption during the Quaternary, which is like basalt islands floating on chalk ocean (Figure 1B; Segev et al., 2002). When the volcano initially erupted about 1 million years ago and cooled down, new vegetation (Hadid et al., 2013), food resources (Figures 1C, 2C), and ecological niches (Figure 1B) emerged, allowing animals to immigrate from the ancestral chalk to the new derivative symparic species on the basalt, forming the new species on the basalt (Hadid et al., 2013; Nevo, 2013; Singaravelan et al., 2013; Li K. et al., 2015; Lövy et al., 2015, 2017, 2020; Li et al., 2016, 2020a,b; Šklíba et al., 2016; Jiao et al., 2021; Mukherjee et al., 2022; Nevo and Li, 2022; Figures 1A, 2C). This provided us with an ideal model to further understand the complex interaction of the gut microbiome, the host, and its corresponding environment.

### The community composition of the gut microbiome between the chalk and basalt sympatric species

Although hundreds of samples are frequently present in many studies, we had only a small number from each population but were still able to demonstrate that richness was not increasing with sample size (Supplementary Figures 3, 8), suggesting the sample size was not a restriction for the analysis. Firmicutes microbes had the largest relative abundance in both chalk and basalt gut microbiome (Figure 1D), which supports evidence found in other studies (Qin et al., 2010; Xiao et al., 2015; Huang et al., 2018). Proteobacteria was significantly higher in the basalt digestion tract (Figure 2D). This particular phylum of bacteria is reported to be positively correlated with the fat intake of the host diet, and is significantly richer in populations with a high-fat diet than that of the malnutritional population (Méndez-Salazar et al., 2018). In this study, the basalt mole rat population was mainly feeding on geophytes (Jiao et al., 2021; Joyce et al., 2022), which have higher fat than that in *Eryngium* sp. roots from chalk; this is congruent with the functional enrichment pathway of fatty acid degradation and Glycerolipid metabolism (Figure 3E) in basalt population. Bacteroidota was higher in chalk than in basalt (Supplementary Figure 6A), which may be caused by the higher fat content in the basalt diet suppressing this phylum (Jeong et al., 2019). If the host is feeding on more proteins and fat, the ratio of Firmicutes/Bacteroidota would be higher (De Filippo et al., 2010). In the present study, the ratio was higher in basalt than in chalk (Supplementary Figure 6B), which may be because of the different host diets primarily geophytes on basalt and primarily roots in the chalk population.

### Functional differentiation between the chalk and basalt gut microbiome

Although the microbiome composition between the two populations is not clearly differentiated (Supplementary Figure 4), the functional compositions showed separated clusters (Figure 2A; Supplementary Figure 9), which may be due to short differentiation time (Supplementary Figure 26) and sharp divergent host diet (Nelson et al., 2013; Lövy et al., 2015; Zmora et al., 2019). Higher functional diversity and abundance of the basalt population (Figures 2B,C) reflect higher food diversity resources in basalt (Hadid et al., 2013). The more abundant (Figures 3F,G) and diverse (Figure 3G; Supplementary Figure 9) CaZymes in chalk populations may be caused by adaptation to poorer food root quality. Mannans are a major component of plant cell walls (La Rosa et al., 2019) and cannot be hydrolyzed by the host itself (Teng and Kim, 2018). We found genes enriched in Mannan binding (GO:2001065; Figure 3D) and catalytic activity (GO:0140097) in chalk, which may be due to higher fiber in the chalk root diet. In basalt populations, the enrichment of transporter activity (GO:0005215), cation transmembrane transport (GO:0022857) and glycogen binding (GO:2001069) reflected better food conditions (Figure 3D). Furthermore, growth (GO:0040007) and oxidoreductase complex (GO:1990204) were also enriched in basalt (Figure 3D), which implies gut microbes play an important role in supplying energy to the host (Tremaroli and Bäckhed, 2012; Zhang et al., 2016). These results correspond to higher metabolic rates and activity rates in basalt populations (Hadid et al., 2013; Zhao et al., 2016). KEGG pathway enrichment analysis also showed similar results (Figure 3E) in basalt, the enrichment of oxidative phosphorylation, photosynthesis and pyruvate metabolism (Figure 3E) revealed basalt populations required more energy and had a higher metabolic rate. The enrichment of fatty acid degradation and glycerolipid metabolism suggested the basalt population consumed a high-fat diet. The metabolic pathways of various other substances (Figure 3E e.g., purine metabolism, thiamine metabolism) also indicated the diversity of basalt food resources. These results suggest that the host diet is a main driver of the gut microbiome divergence.

### Genetic divergence of the gut and soil microbiome between the chalk and basalt

The divergence of soil microbiome between the chalk and basalt is larger than gut microbiome. At the species level (Mukherjee et al., 2022), we found that the soil bacteria differentiation between basalt and chalk is larger than that of the same species in the gut (Figure 4D), which may be due to a combination of more contrasting edaphic stresses and longer divergence time of the soil microbiome. The divergence of the soil microbiome started when volcanic eruptions formed new habitats 1 million years ago, but the hosts of blind mole rats split much later only 0.228 Mya which hampered the gut microbial divergence (Supplementary Figure 4). As the bacteria from the same host individual are under the same intestine stresses, we can expect that the effects of host on the gut microbiome are similar. However, some species displayed significant divergence between basalt and chalk (Supplementary Figures 2, 12–33), which also echoed the results above (Figures 4A,C; Supplementary Figures 12–31).

### Protein divergence of the hosts mole rats populations

Generally, the host blind mole rats showed separate clusters in protein between the basalt and chalk populations (Figure 5B). We identified 127 significantly differential proteins (Figure 5C). The enrichment results showed that these differential proteins were involved in the metabolism and synthesis of a wide range of species (Supplementary Figure 34) and were mainly secreted proteins (Supplementary Figure 35). These results correspond to the different diets of the two populations. One individual from the basalt population was clustered into the chalk group, possibly due to the proteome differentiating more slowly than the genome.

### The major factor that shaped the gut microbiome

The community composition between the chalk and basalt gut microbiome was not significantly different (Supplementary Figure 4) when compared to its host phylogeny (Figure 1D), host diet (Figure 2) or the environmental soil metagenomes (Mukherjee et al., 2022), which may be due to the fact that divergence between the chalk and basalt microbiome occurred much later (Li K. et al., 2015). However, the main question still stands: which environmental factor is the main driver of the microbial divergence between the chalk and basalt, or have all of them shaped the gut microbiome together? For host phylogeny, we showed that the two mole rats populations diverged in the genome (Li K. et al., 2015), methylome (Li et al., 2020b), transcriptome, and genomic editing (Li et al., 2016) and even the proteins (Figure 5B,C; Supplementary Figures 34, 35). If the gut microbiome were shaped mainly by host phylogeny and changed synchronously, we would expect the host phylogeny to mirror that of the gut microbiome (Brooks et al., 2016; Perofsky et al., 2019). However, we found that the host phylogeny was not consistent with the gut microbiome (Figure 5A). Additionally, the mantel test showed there were no correlations (r = 8.078e-3, *p* = 0.4528) between the host genetic matrix and Bray-Curtis distance matrix based on the gut microbiome; this may be due to the short differentiation time of the gut microbiome (Supplementary Figures 12B, 13C–31C), or that the host phylogeny does not affect it. The significant differences in functional composition (Figure 3) illustrates that the gut microbiome had adapted to the host’s local ecology which is dramatically different between the calcareous chalk and siliceous basalt (Figures 3D–G; Supplementary Figures 9–11). Basically, the same species or similar species compositions in the gut microbiome of blind mole rats can perform different functions, which was consistent with our finding of differentiation of the probably same species between the two types of environments (Figure 4; Supplementary Figures 12–31). The microbe from the two contrasting soils was significantly different in both community composition and function (Mukherjee et al., 2022). The divergence of the gut microbiome was significantly smaller than that of the soil microbiome (Figure 4); this is likely because the soil difference is larger than that of the gut environment. Another possibility that should be explored in the future is that the bacteria in the microbiome underwent sympatric speciation following their hosts, This possibility should be studied in the future in representative dominant bacteria in the microbiome of both hosts.The chalk and basalt rocks and soils are abutting but contrasting with different chemicals (Grishkan et al., 2008, 2009). The community composition of the soil is significantly different from that of the gut (Figure 2A). Both the procruster analysis (Figure 2B) and mantel test (r = −0.01518, *p* = 0.5317) showed there was no significant correlation between the soil and gut microbiome, allowing us to reject the hypothesis that the local environment was the main factor shaping the gut microbiome.

The food store for *S. galili* chalk is from *Eryngium* sp., poaceae roots, and *Ranunculus sp.* leaves; while the *S. galili* basalt mainly feed on geophytes, including *Hordeum bulbosum*, *Bellevalia sp.*, *Iris histrio,* and a very small amount of *Eryngium sp.* (Jiao et al., 2021)*.* The geophytes from the basalt are rich in protein and fat, while it is rich in cellulose in bushlets roots in chalk (Lövy et al., 2015, 2017). The derivative *S. galili* basalt split from its ancestral *S. galili* chalk, and so does the gut microbiome, which may explain why there was no significant differences in species diversity and composition (Supplementary Figures 4, 5). Unless bacteria also underwent sympatric speciation like their hosts, a major issue that should be explored in the future. Nevertheless, in terms of species and function such as Proteobacteria (Figure 2D) and CAZymes composition, characteristics of the gut microbiome adapted to their host’s diet were also found. Differences in diet may also be involved in the divergence of blind mole rats (Martin, 2013; Nelson et al., 2013; Li K. et al., 2015; Ekimova et al., 2019). SS of the hosts, blind mole rat, and functional adaptation rather than the composition of the gut microbiome were expected to be synchronized. Additionally, here we demonstrated that function or species composition of the gut microbiome are evolving to adapt to their local ecology dramatically different between chalk and basalt ecologiest (Figures 1E, 2C,D, 3A, 4A). Although we can make a preliminary conclusion that host diet is the main driver of functional divergence of the gut microbiome, further experimental tests are required to preclude the roles of phylogeny and environment on the gut microbiome differentiation. Likewise as indicated above the likely possibility of sympatric speciation of the gut microbiome followed that of their hosts.

## Conclusion

We performed gut metagenomic comparison between the two populations, and correlate the differentiation with its corresponding environment, host diet and phylogeny. No significant differences were found in species composition of gut microbes between Basalt and Chalk, but were found in some phyla such as *Proteobacteria*, corresponding to their different host diets. Significant differences were also detected at the strain level between the two species populations. We found significant differences in the functional composition, which was due to the adaptation of gut microbiome to different diets. The gut microbiome does not drive the host separation, vise versa. In addition, we found no significant association between host genetics, soil microbiomes and gut microbiomes. We demonstrated that function or species composition of the gut microbiome are evolving to adapt to their local environment, primarily diet content.

## Data availability statement

The datasets presented in this study can be found in online repositories. The names of the repository/repositories and accession number(s) can be found at: https://www.ncbi.nlm.nih.gov/, PRJNA876956.

## Ethics statement

This study was approved by Ethics Committee of College of Ecology, Lanzhou University and in accordance with the current ethical review: Complies with the ethical requirements and agree to study in accordance with this scheme (No. EAF2022013).

## Author contributions

EN and KL designed the research. ZK, FL, QD, and CT performed the research. ZK, EN, and KL wrote the paper. All authors contributed to the article and approved the submitted version.

## Funding

This project was supported by the National Natural Science Foundation of China (32271691 and 32071487), National Key Research and Development Programs of China (2021YFD1200901), Science Fund for Creative Research Groups of Gansu Province (21JR7RA533), Lanzhou University’s “Double First-Class” Guided Project-Team Building Funding-Research Startup Fee for KL, Chang Jiang Scholars Program, The Fundamental Research Funds for Central Universities, LZU (lzujbky-2021-ey17), a grant from State Key Laboratory of Grassland Agro-Ecosystems (Lanzhou University; Grant Numbers: SKLGAE-202001, 202009, and 202010), Ancell-Teicher Research Foundation for Genetic and Molecular Evolution for its constant financial support for supporting *Spalax* research Program. We received support for computational work from the Big Data Computing Platform for Western Ecological Environment and Regional Development and Supercomputing Center of Lanzhou University.

## Conflict of interest

The authors declare that the research was conducted in the absence of any commercial or financial relationships that could be construed as a potential conflict of interest.

## Publisher’s note

All claims expressed in this article are solely those of the authors and do not necessarily represent those of their affiliated organizations, or those of the publisher, the editors and the reviewers. Any product that may be evaluated in this article, or claim that may be made by its manufacturer, is not guaranteed or endorsed by the publisher.

## Footnotes

^1^ https://rdrr.io/cran/vegan/

^2^ http://huttenhower.sph.harvard.edu/galaxy/

^3^ https:/www.cloudtutu.com

^4^ https://github.com/wwood/CoverM

^5^ https://github.com/jameslz/biostack-suits
