# Supplementary material for: Host diet shapes functionally differentiated gut microbiomes in sympatric speciation of blind mole rats in Upper Galilee, Israel
Source: Front Microbiol. 2022 Nov 15;13:1062763. doi: 10.3389/fmicb.2022.1062763 (PMC9707624; doi:10.3389/fmicb.2022.1062763)
Supplement: Supplementary file 1 [file Data_Sheet_1.docx]

Supplementary Material


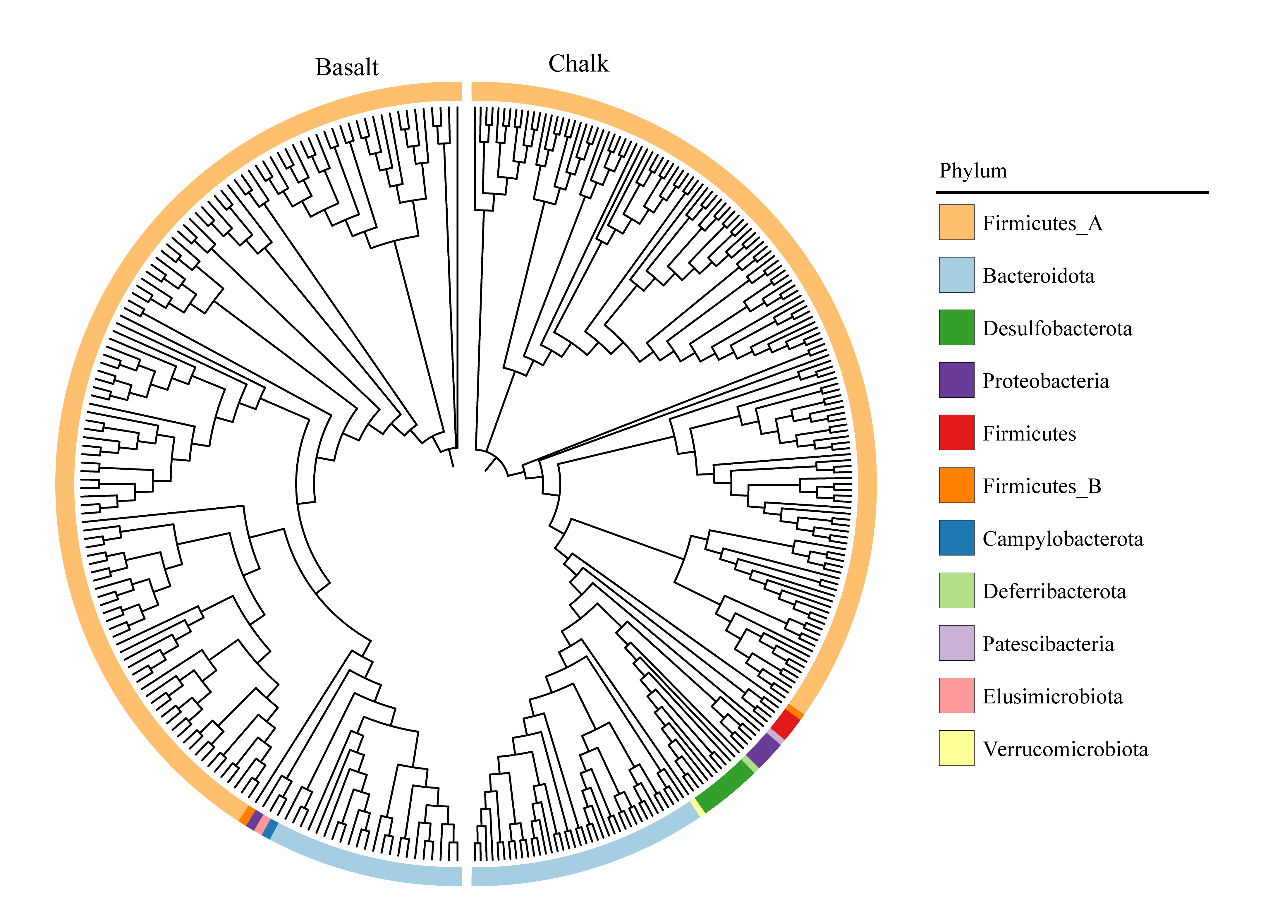


**Supplementary Figure 1.** Phylogenetic relationships and taxonomic classifications of the 339 bins from basalt gut and chalk gut.


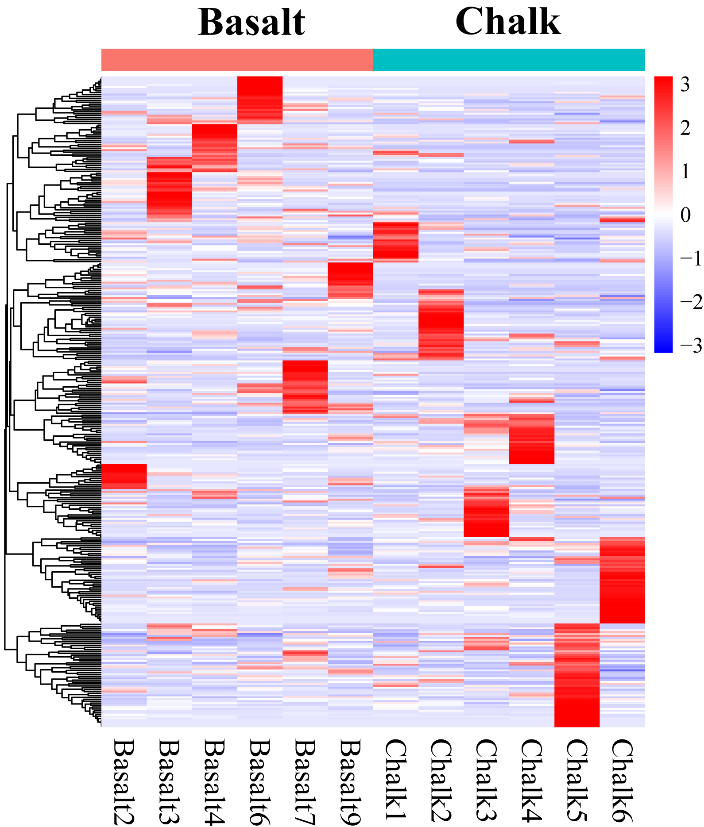


**Supplementary Figure 2.** Heatmap based on relative abundance of MAGs.


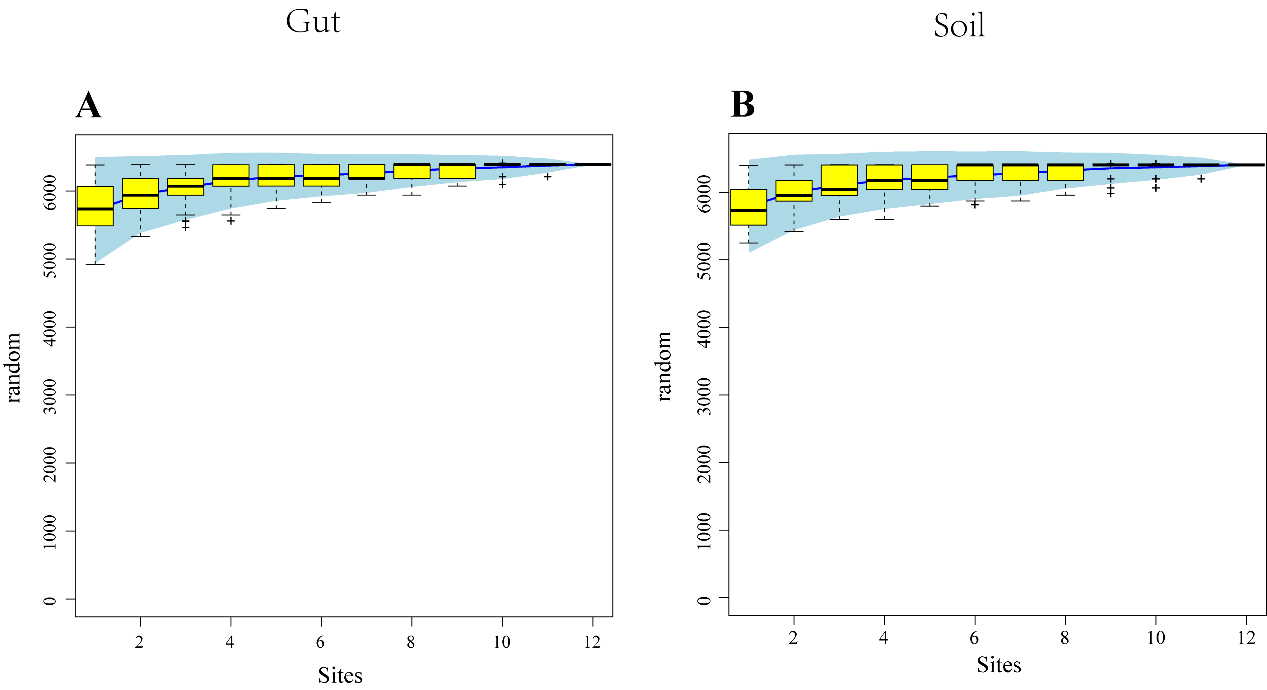


**Supplementary Figure 3.** Species accumulation curve. (A) Species accumulation curve of gut samples. (B) Species accumulation curve of soil samples. The horizontal coordinate is the number of samples and the vertical coordinate is the number of species found.


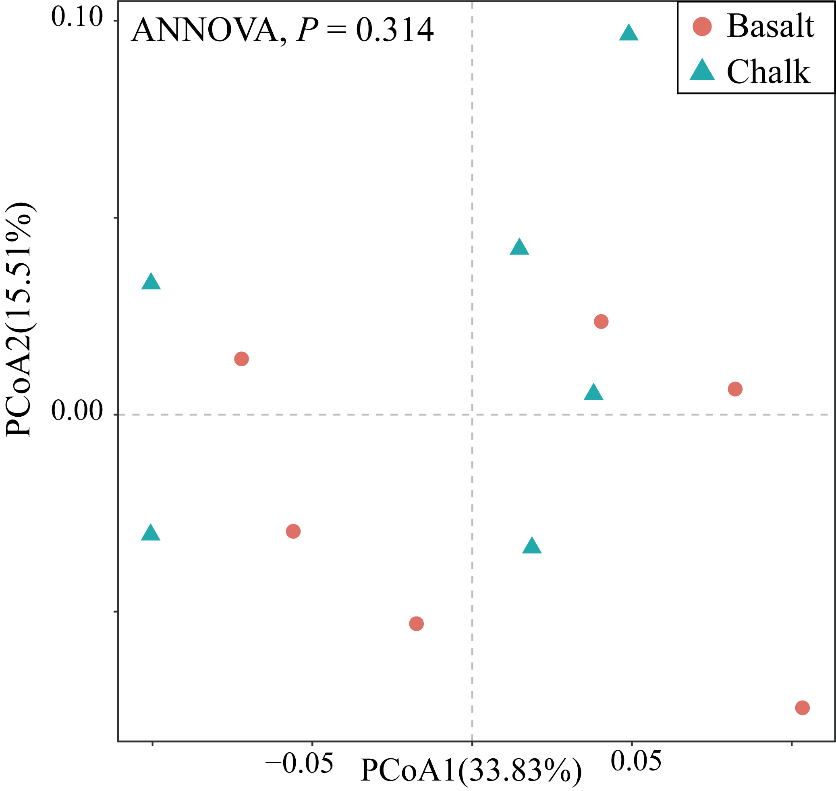


**Supplementary Figure 4.** Principal component analysis showed the species composition was not significantly separated.


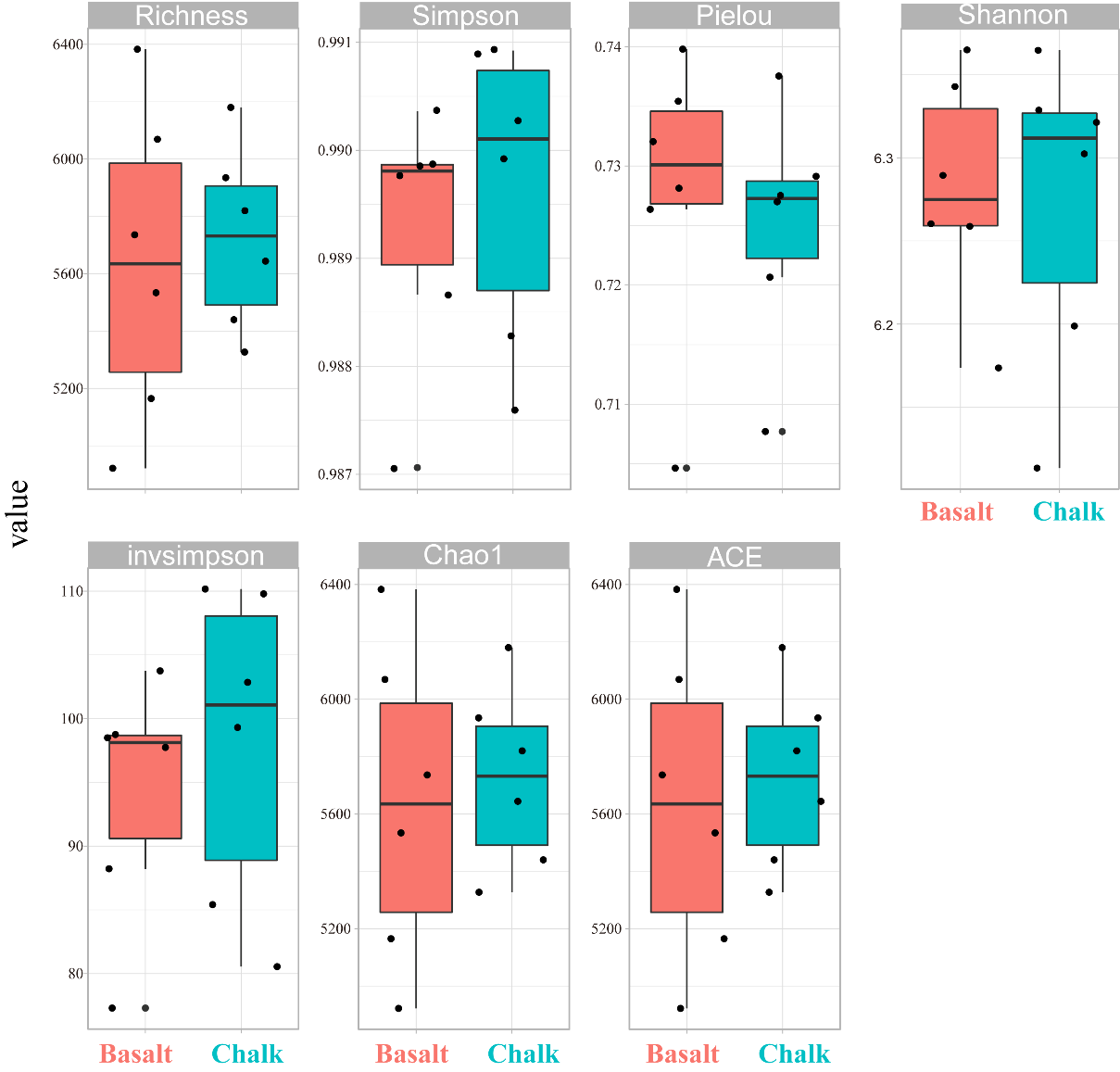


**Supplementary Figure 5.** Various alpha diversity calculated based on gut microbiome composition.

**
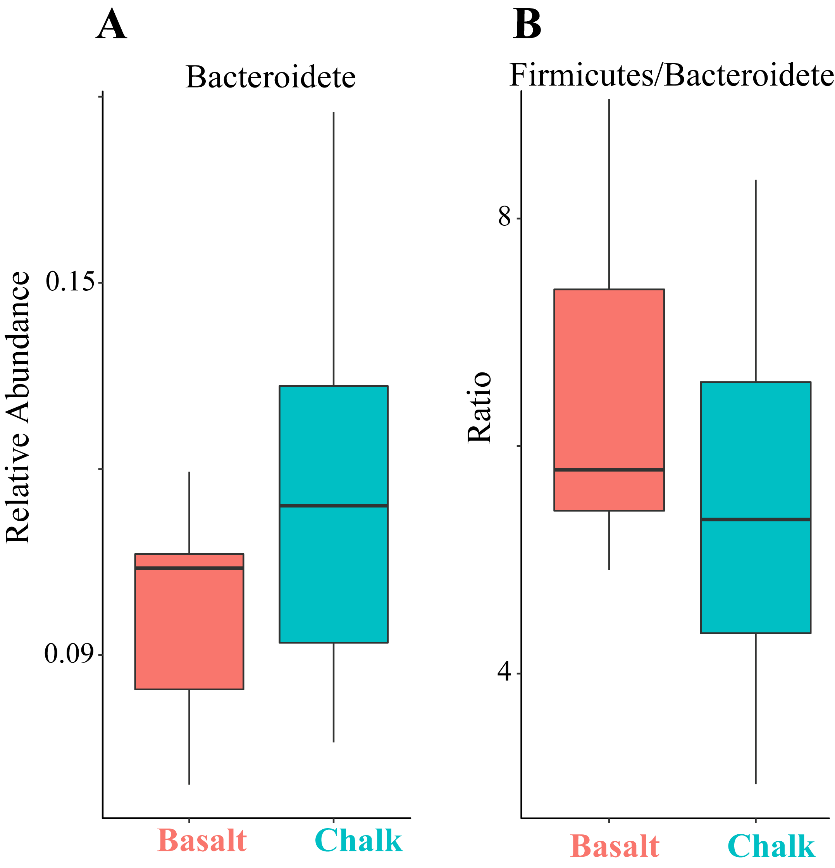
**

**Supplementary Figure 6.** (A) Bacteroidete was more abundant in chalk. (B) The ratio of Firmicutes to Bacteroidetes (F/B ratio) was higher in basalt.


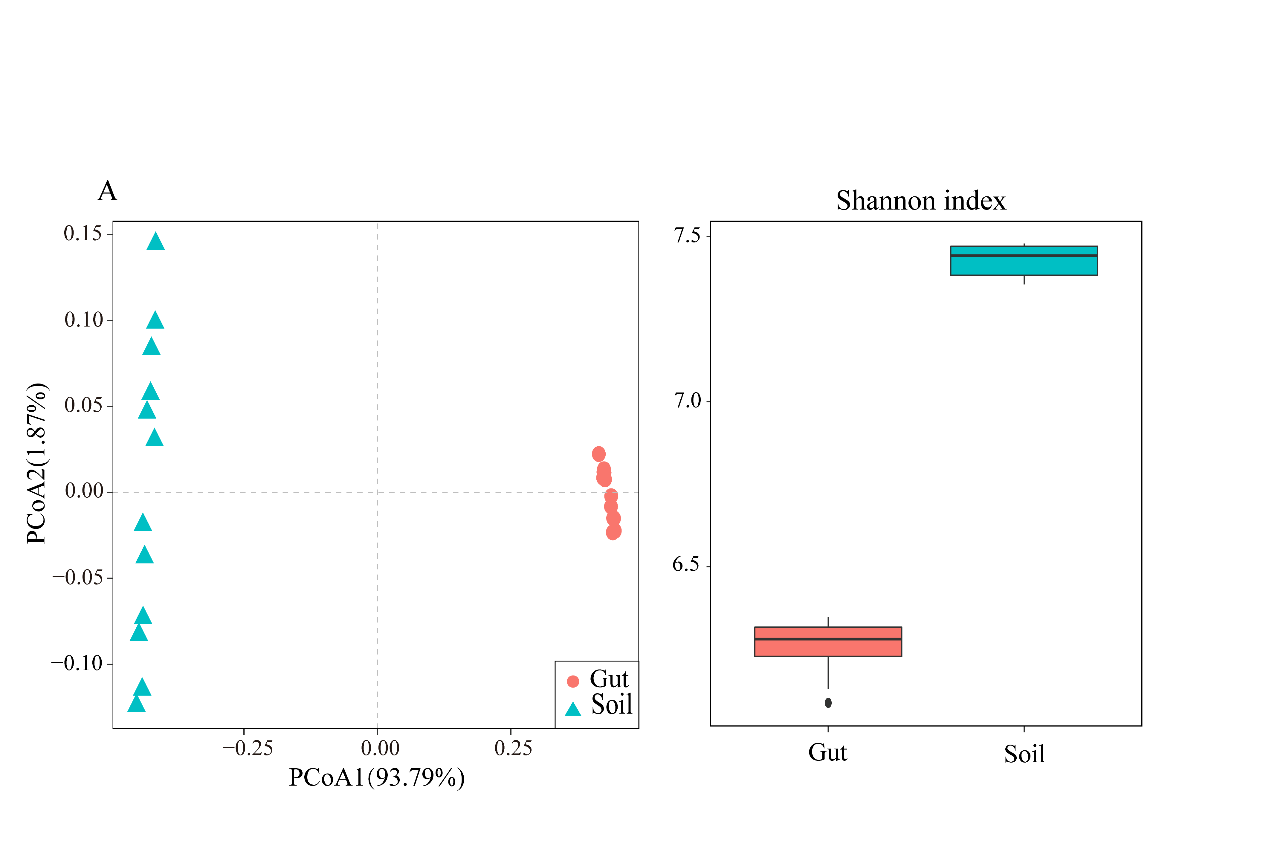


**Supplementary Figure 7.** Soil microbiome had a significantly higher Shannon index than gut microbiome.


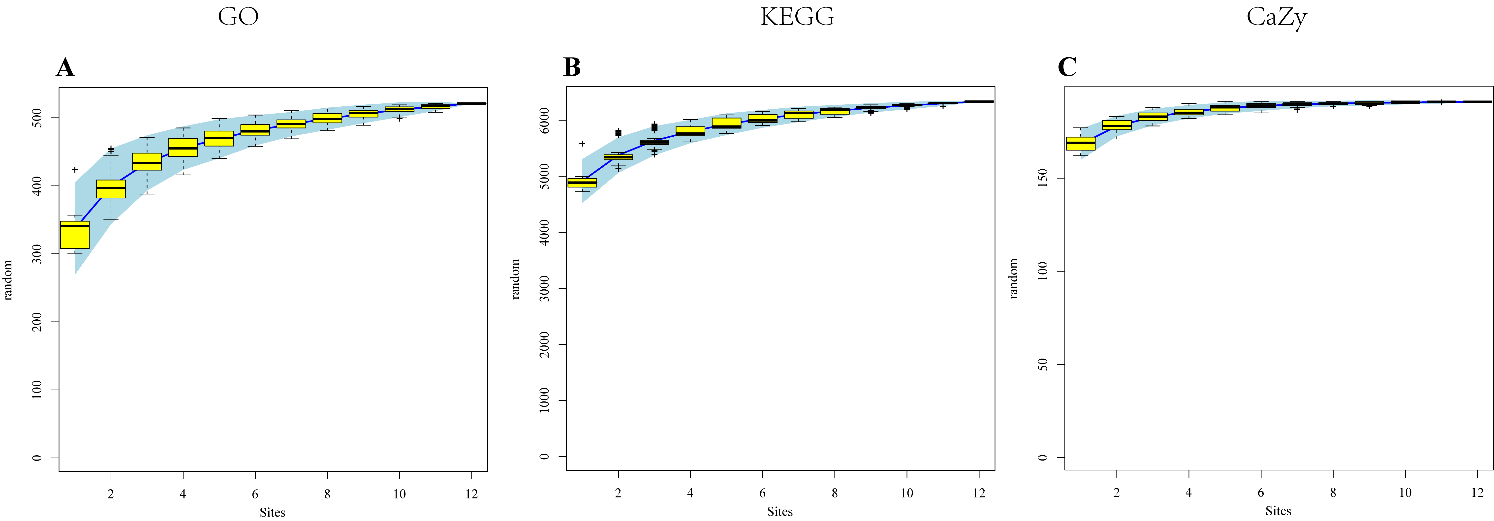


**Supplementary Figure 8.** Cumulative curves for (A) GO, (B) KEGG and (C) CaZy.


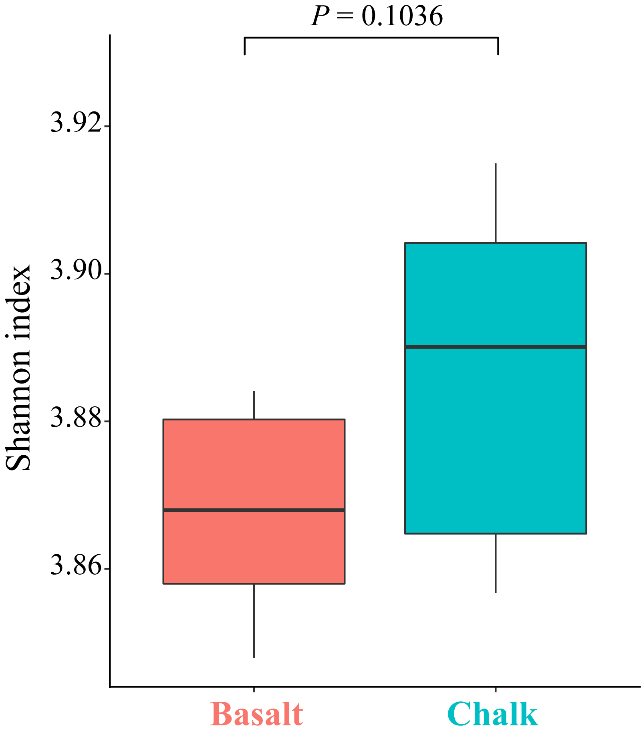


**Supplementary Figure 9.** Higher carbohydrate active enzymes (CAZyomes) shannon diversity in chalk.


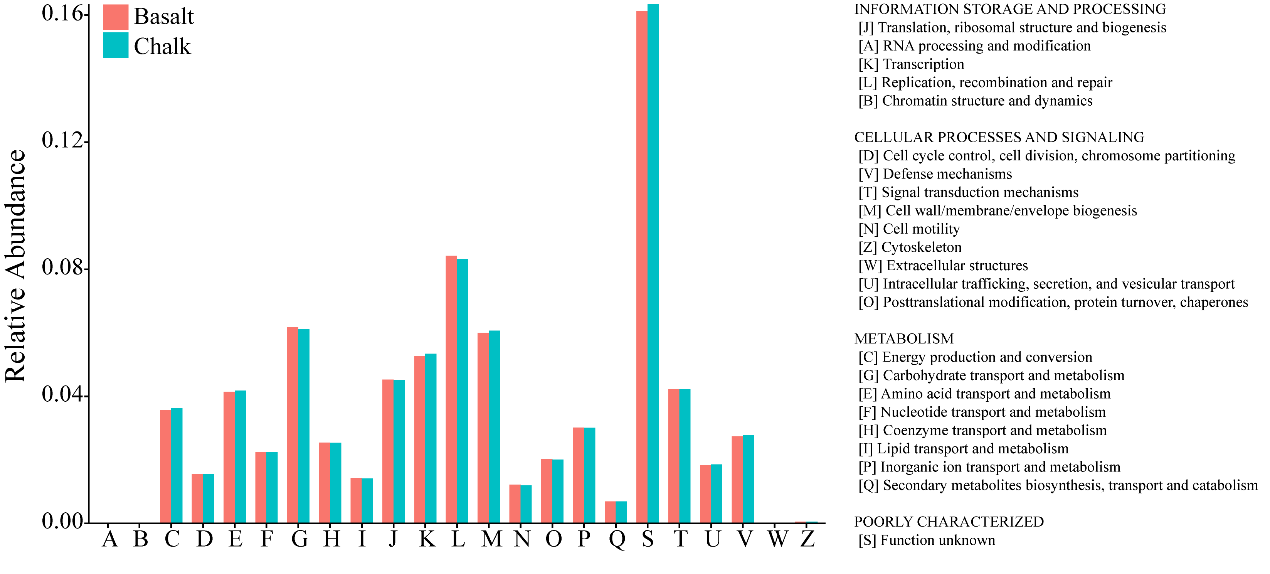


**Supplementary Figure 10.** Functional categories for COGs based on non-redundant gene catalog.


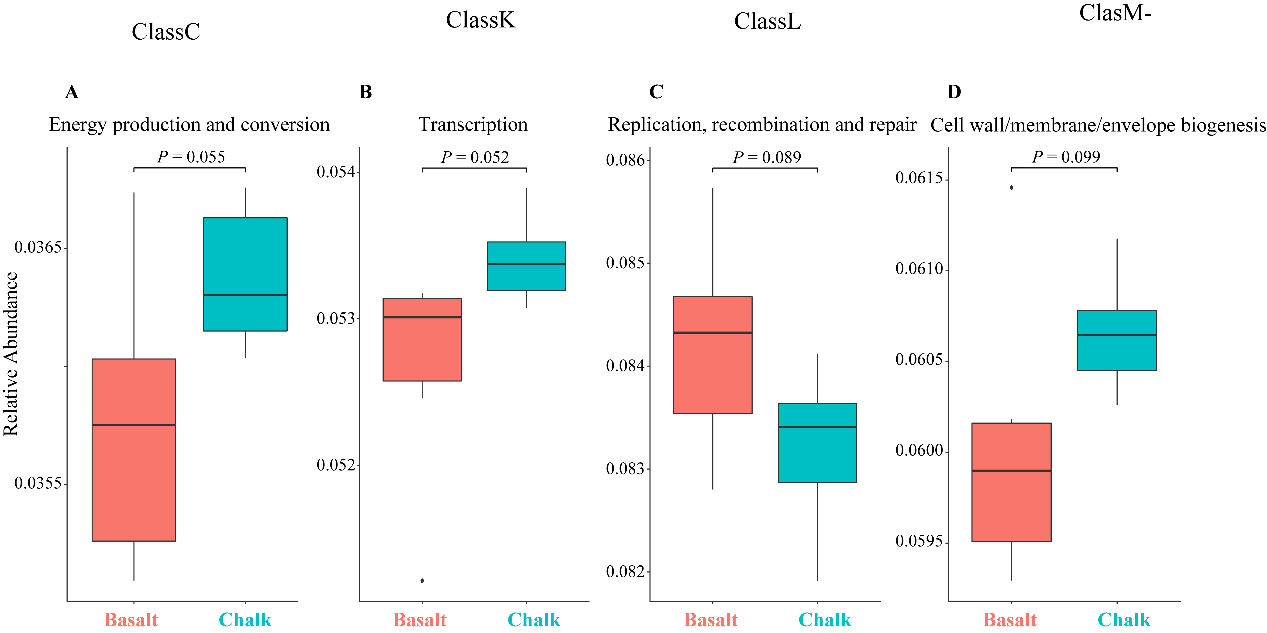


**Supplementary Figure 11.** COG classes with differences in relative abundance.


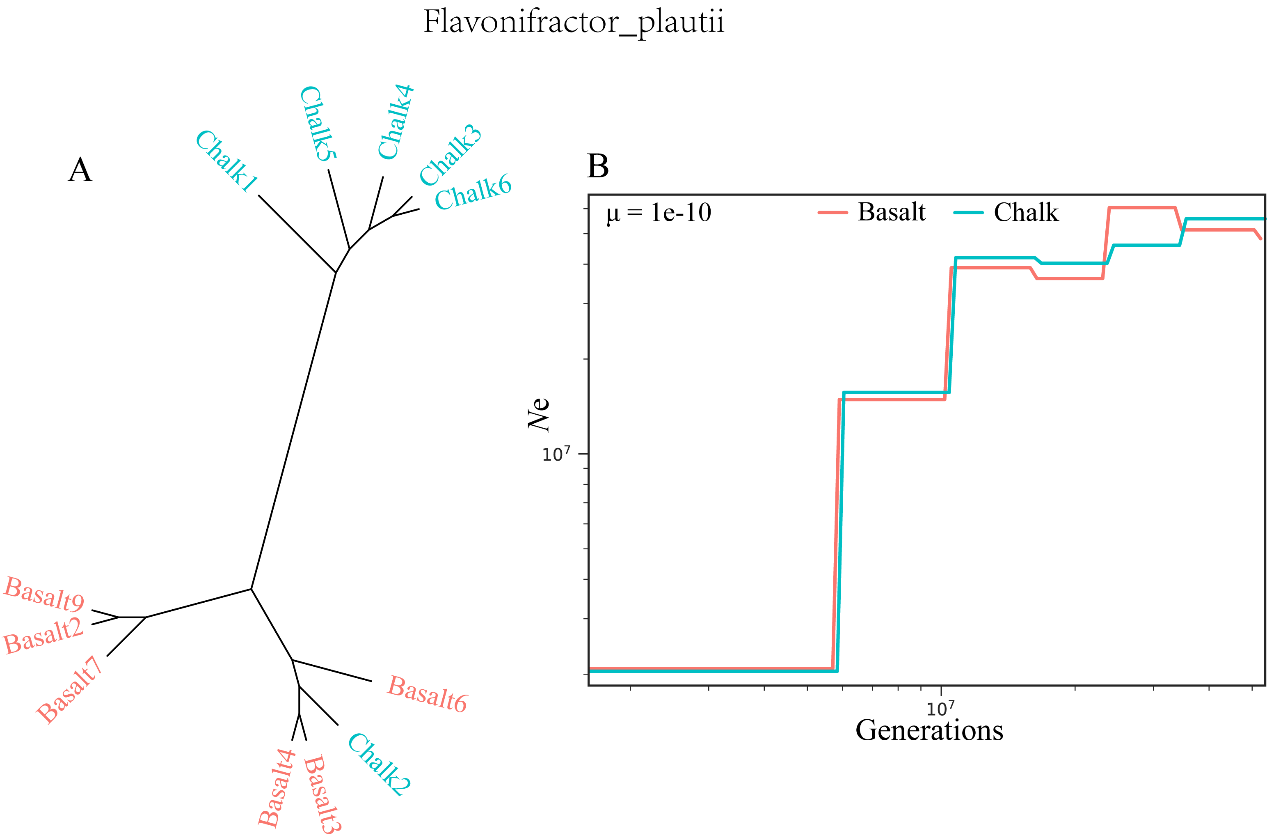


**Supplementary Figure 12.** Sympatric divergence of the single bacteria of *Flavonifractor plautii*. (A) Phylogenetic tree of the chalk and abutting basalt populations. (B) Population demographic history.


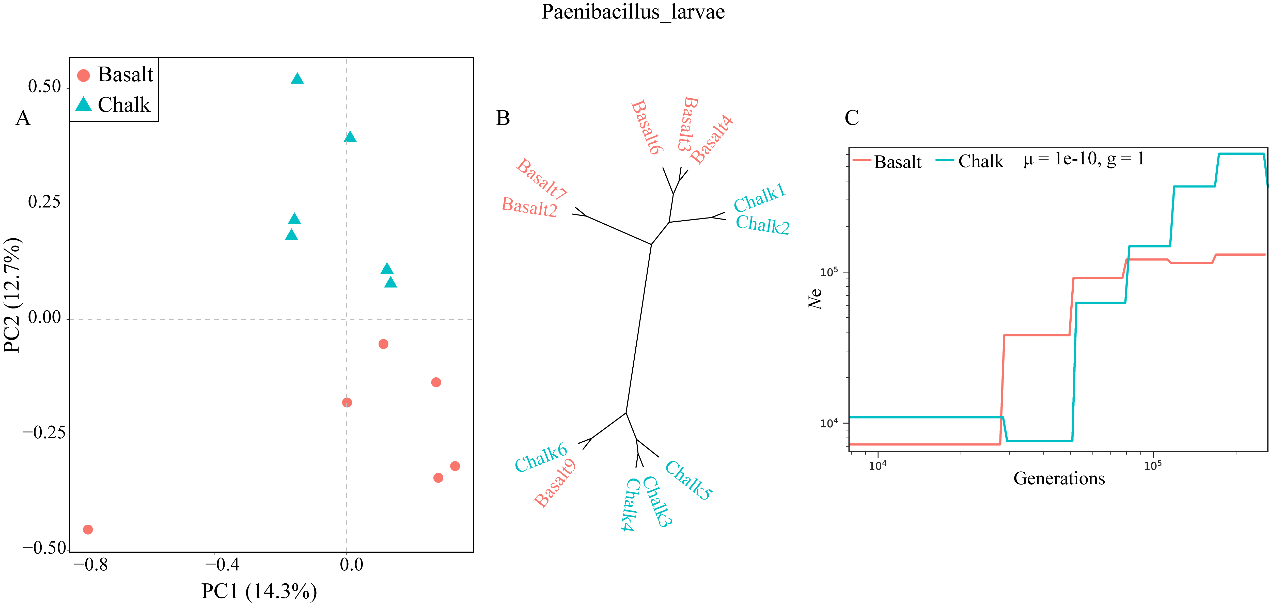


**Supplementary Figure 13.** Sympatric divergence of the single bacteria of *Paenibacillus larvae*. (A) Principal component analysis shows samples from basalt clustered together and samples from chalk were in one cluster. (B) Phylogenetic tree of the chalk and abutting basalt populations. (C) Population demographic history.


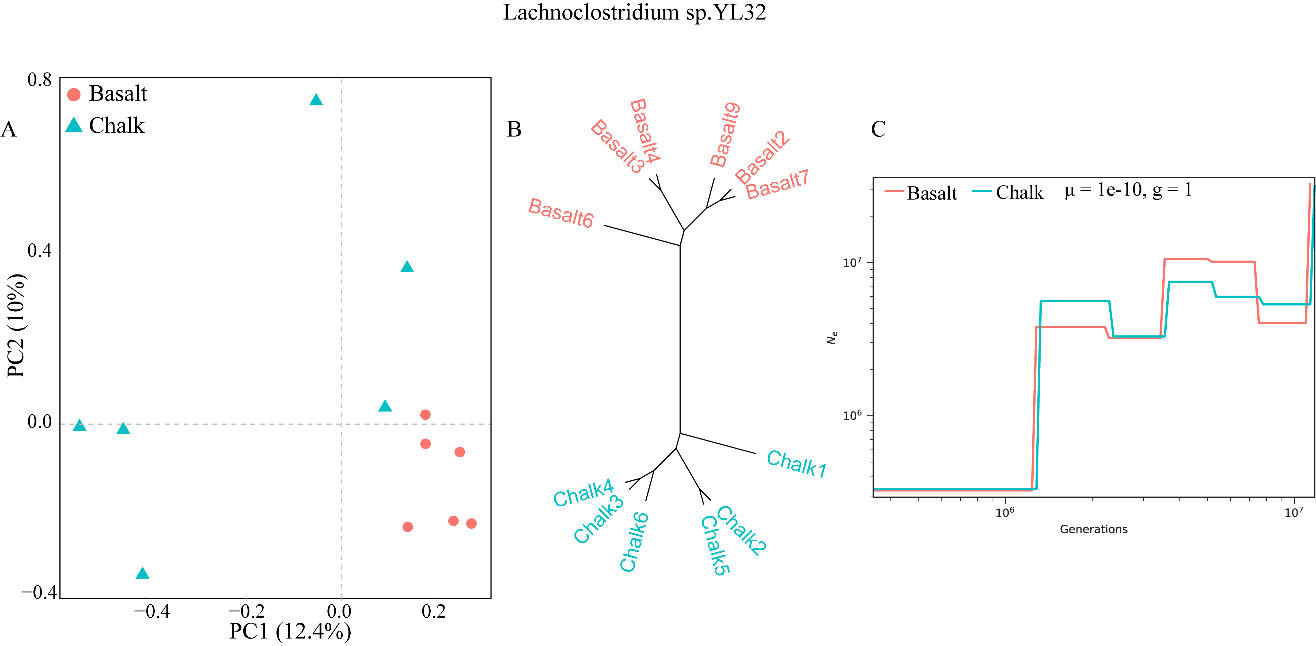


**Supplementary Figure 14.** Sympatric divergence of the single bacteria of *Lachnoclostridium* sp. YL32. (A) Principal component analysis shows samples from basalt clustered together and samples from chalk were in one cluster. (B) Phylogenetic tree of the chalk and abutting basalt populations. (C) Population demographic history.


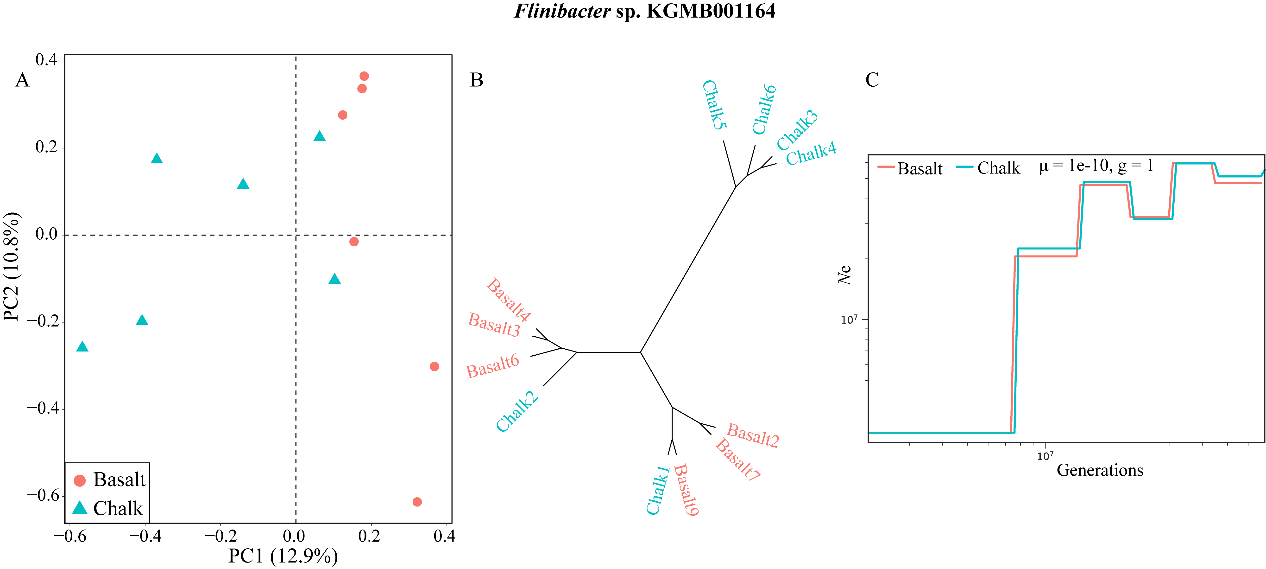


**Supplementary Figure 15.** Sympatric divergence of the single bacteria of *Flinibacter* sp. KGMB001164. (A) Principal component analysis shows samples from basalt clustered together and samples from chalk were in one cluster. (B) Phylogenetic tree of the chalk and abutting basalt populations. (C) Population demographic history.


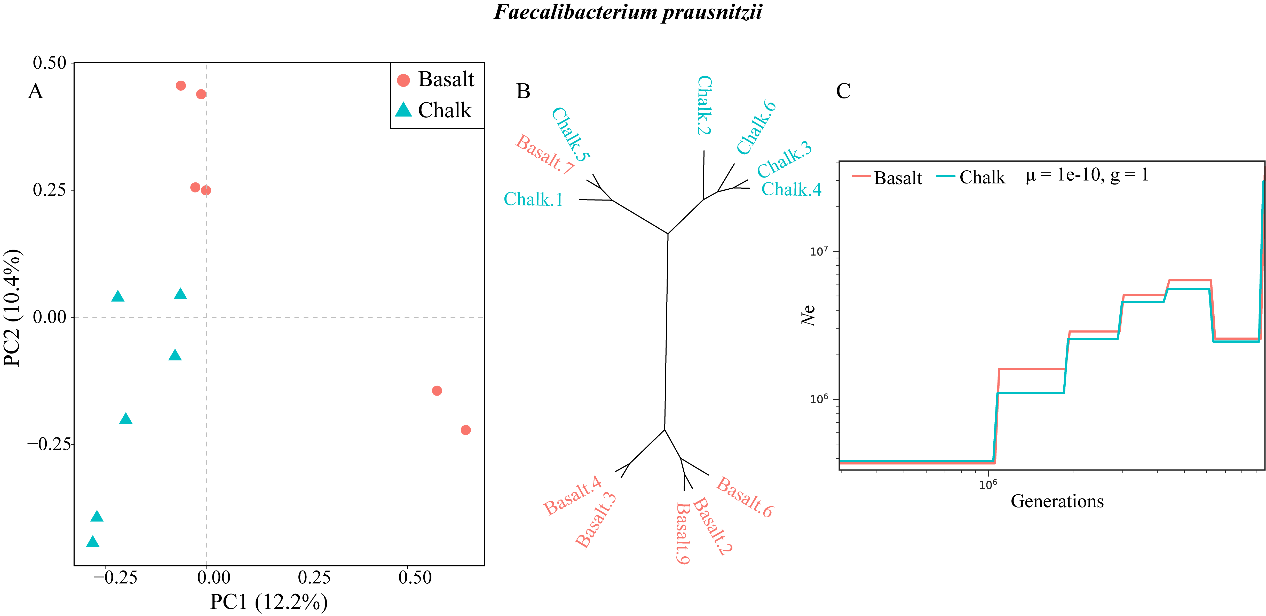


**Supplementary Figure 16.** Sympatric divergence of the single bacteria of *Faecalibacterium prausnitzii*. (A) Principal component analysis shows samples from basalt clustered together and samples from chalk were in one cluster. (B) Phylogenetic tree of the chalk and abutting basalt populations. (C) Population demographic history.


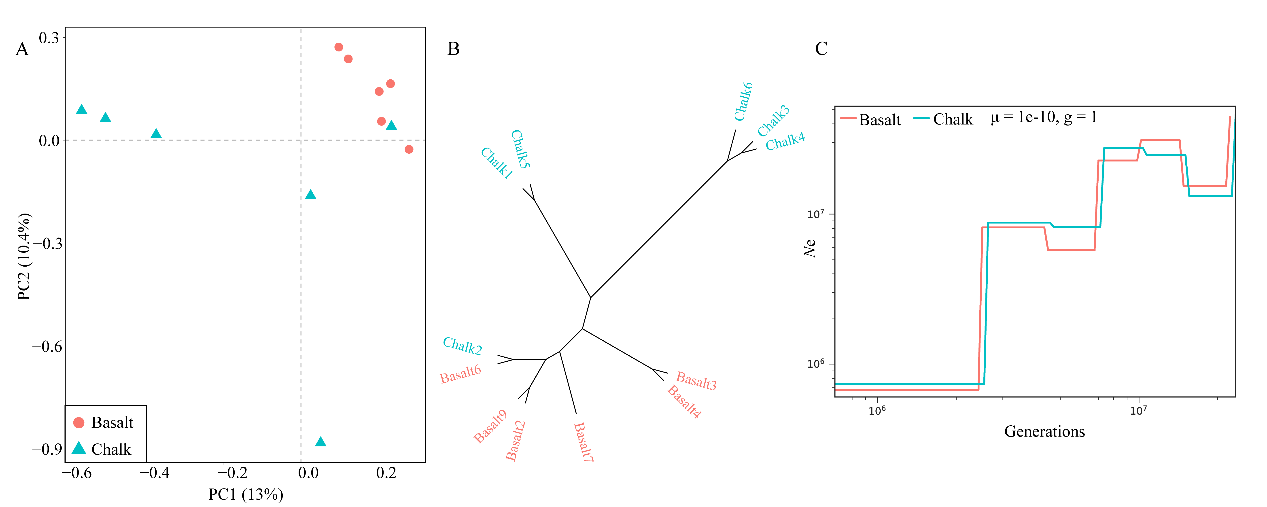


**Supplementary Figure 17.** Sympatric divergence of the single bacteria of *Acutalibacter muris*. (A) Principal component analysis shows samples from basalt clustered together and samples from chalk were in one cluster. (B) Phylogenetic tree of the chalk and abutting basalt populations. (C) Population demographic history.


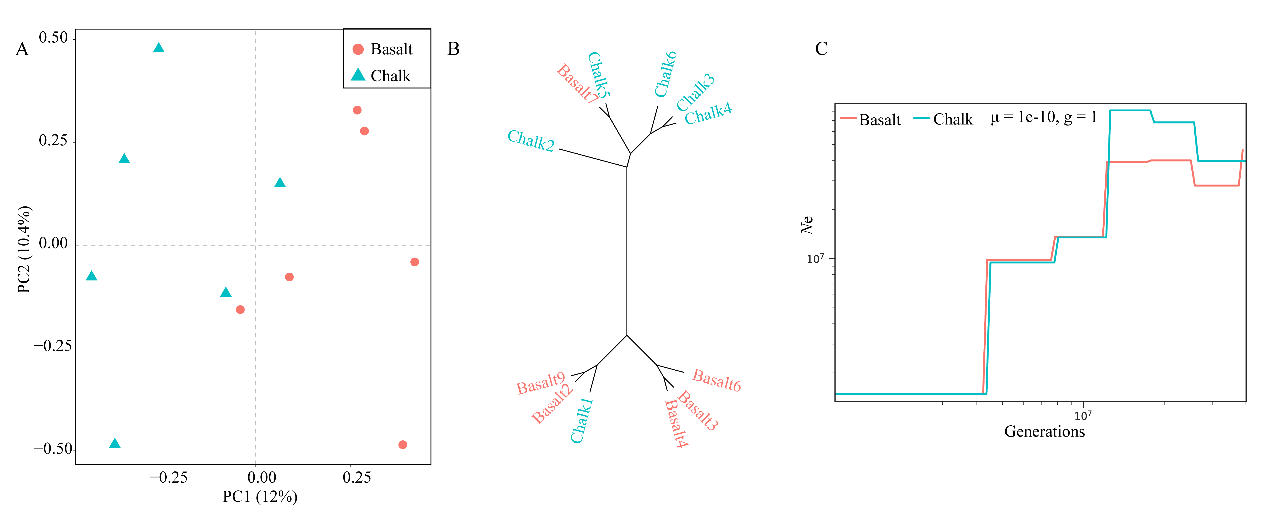


**Supplementary Figure 18.** Sympatric divergence of the single bacteria of *Oscillibacter* sp. NSJ-62. (A) Principal component analysis shows samples from basalt clustered together and samples from chalk were in one cluster. (B) Phylogenetic tree of the chalk and abutting basalt populations. (C) Population demographic history.


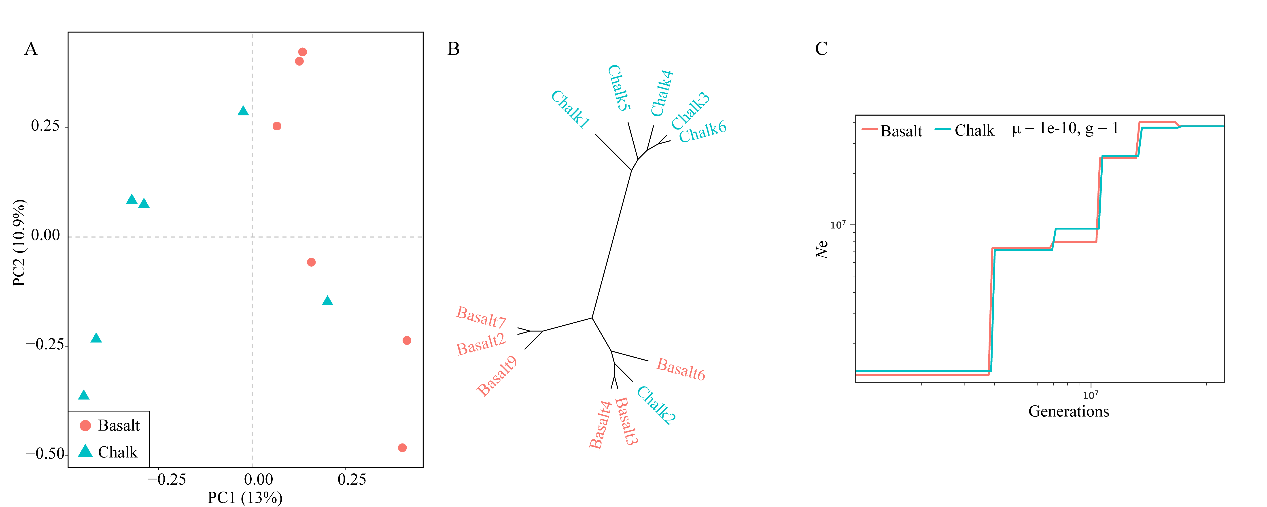


**Supplementary Figure 19.** Sympatric divergence of the single bacteria of *Intestinimonas butyriciproducens*. (A) Principal component analysis shows samples from basalt clustered together and samples from chalk were in one cluster. (B) Phylogenetic tree of the chalk and abutting basalt populations. (C) Population demographic history.


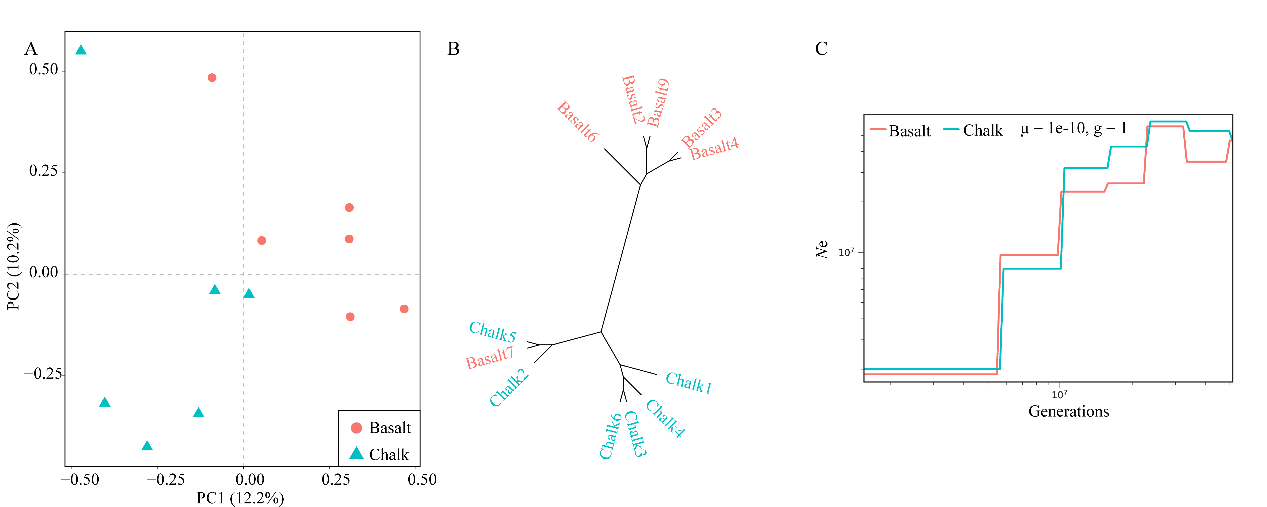


**Supplementary Figure 20.** Sympatric divergence of the single bacteria of *Dysosmobacter welbionis*. (A) Principal component analysis shows samples from basalt clustered together and samples from chalk were in one cluster. (B) Phylogenetic tree of the chalk and abutting basalt populations. (C) Population demographic history.


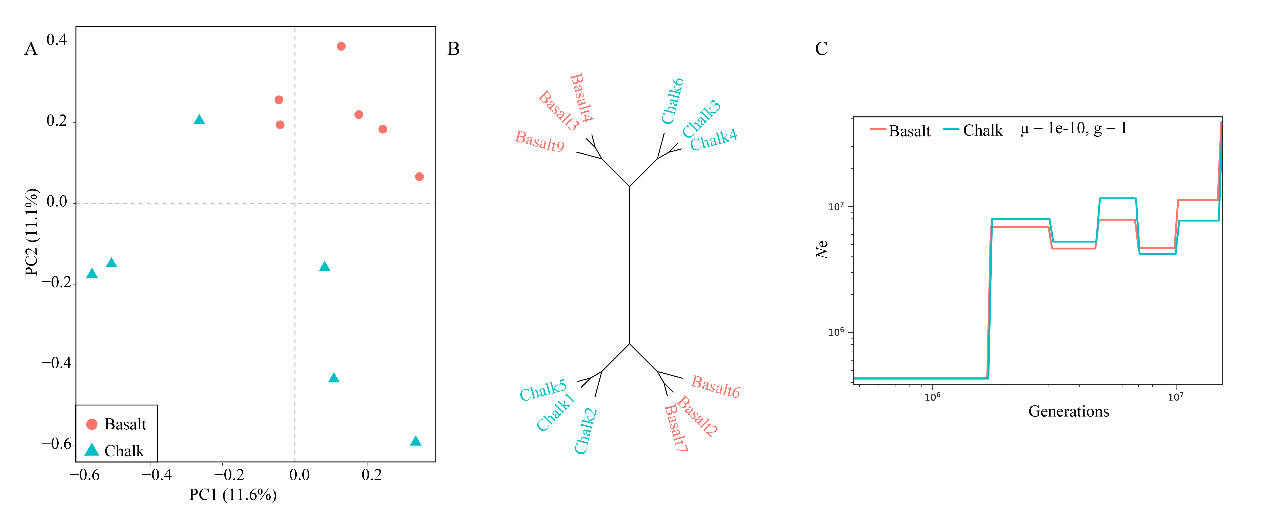


**Supplementary Figure 21.** Sympatric divergence of the single bacteria of*Lachnoclostridium phocaeense*. (A) Principal component analysis shows samples from basalt clustered together and samples from chalk were in one cluster. (B) Phylogenetic tree of the chalk and abutting basalt populations. (C) Population demographic history.


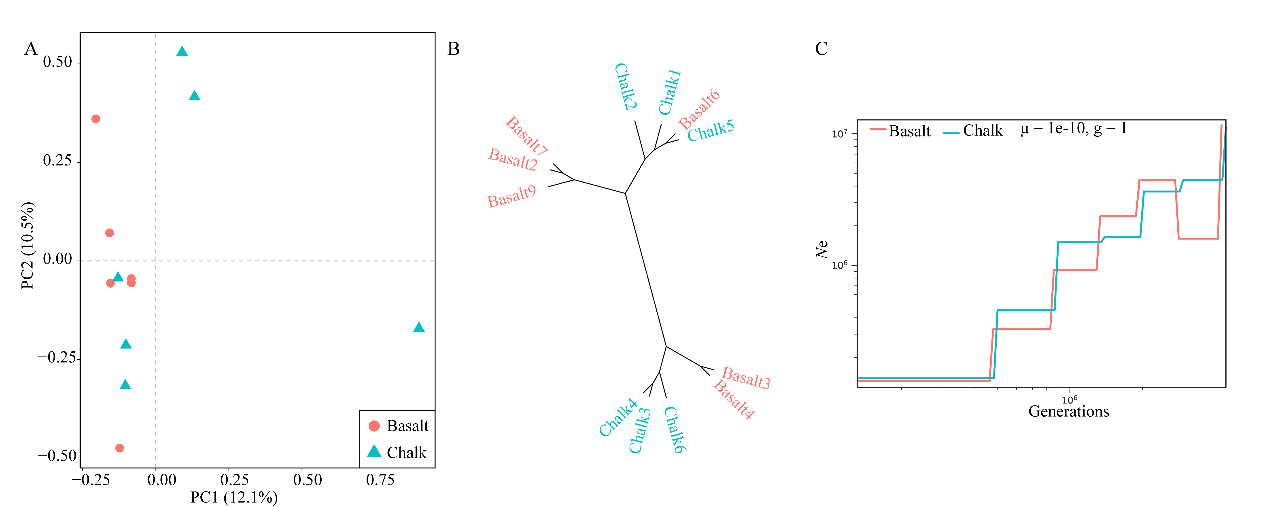


**Supplementary Figure 22.** Sympatric divergence of the single bacteria of *Blautia producta*. (A) Principal component analysis shows samples from basalt clustered together and samples from chalk were in one cluster. (B) Phylogenetic tree of the chalk and abutting basalt populations. (C) Population demographic history.


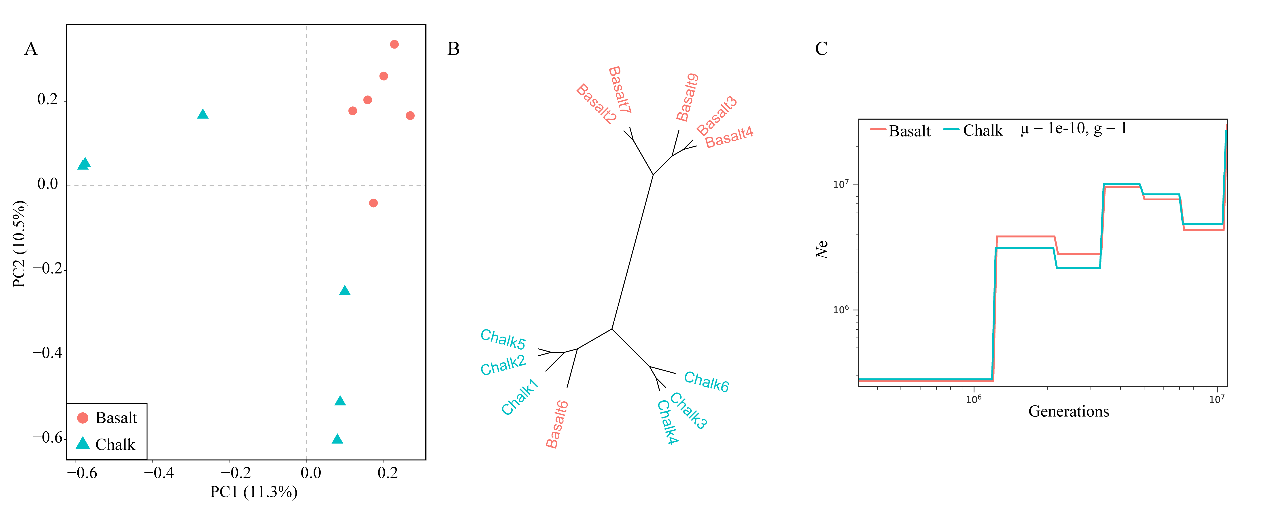


**Supplementary Figure 23.** Sympatric divergence of the single bacteria of *Roseburia intestinalis*. (A) Principal component analysis shows samples from basalt clustered together and samples from chalk were in one cluster. (B) Phylogenetic tree of the chalk and abutting basalt populations. (C) Population demographic history.


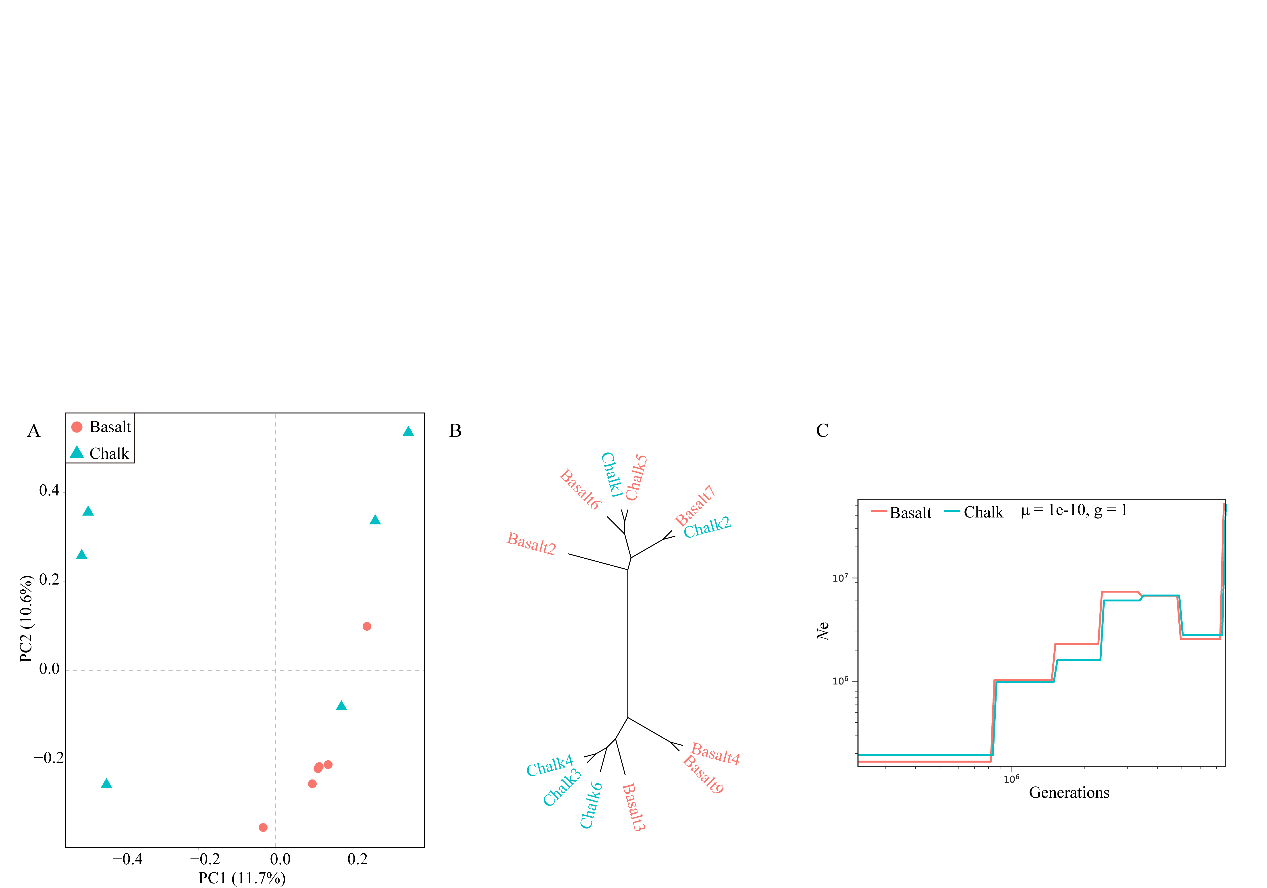


**Supplementary Figure 24.** Sympatric divergence of the single bacteria of *Enterocloster bolteae*. (A) Principal component analysis shows samples from basalt clustered together and samples from chalk were in one cluster. (B) Phylogenetic tree of the chalk and abutting basalt populations. (C) Population demographic history.


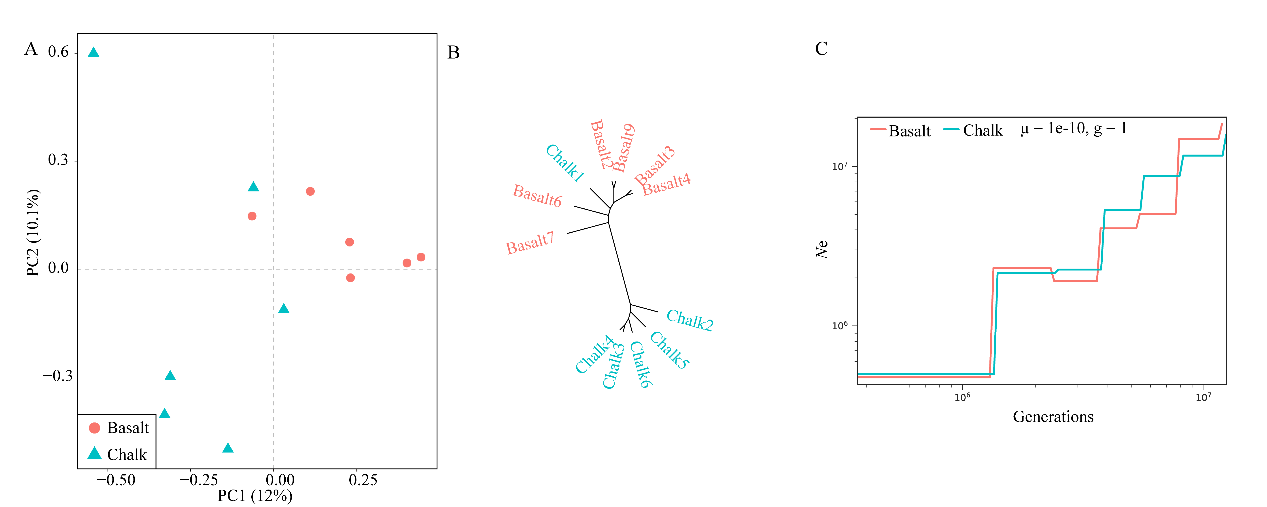


**Supplementary Figure 25.** Sympatric divergence of the single bacteria of *Oscillibacter valericigenes*. (A) Principal component analysis shows samples from basalt clustered together and samples from chalk were in one cluster. (B) Phylogenetic tree of the chalk and abutting basalt populations. (C) Population demographic history.


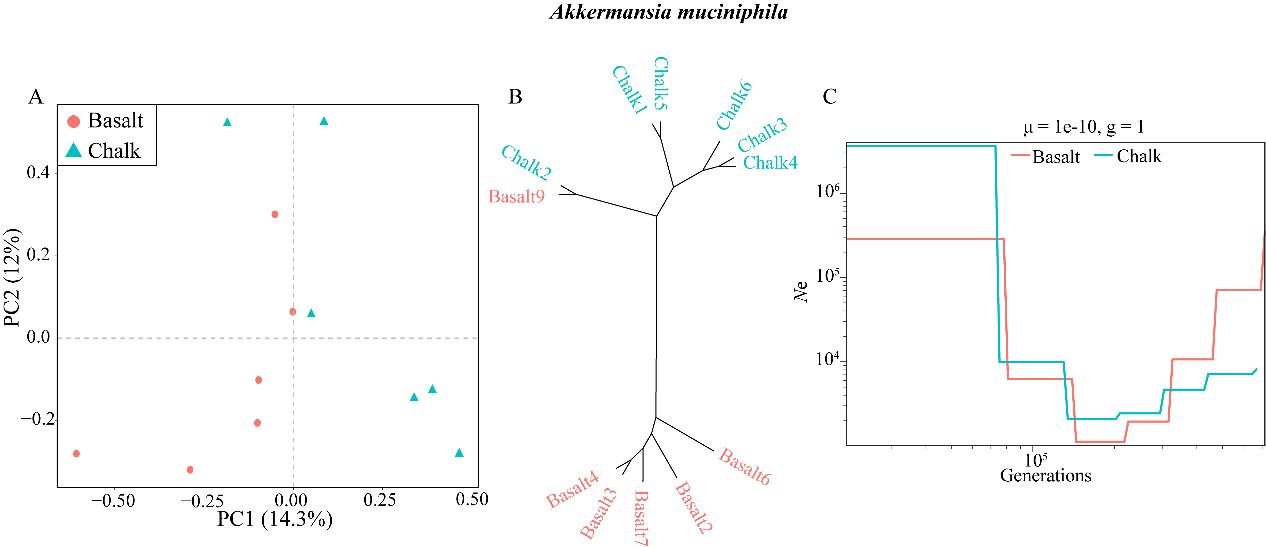


**Supplementary Figure 26.** Sympatric divergence of the single bacteria of *Akkermansia muciniphila*. (A) Principal component analysis shows samples from basalt clustered together and samples from chalk were in one cluster. (B) Phylogenetic tree of the chalk and abutting basalt populations. (C) Population demographic history.


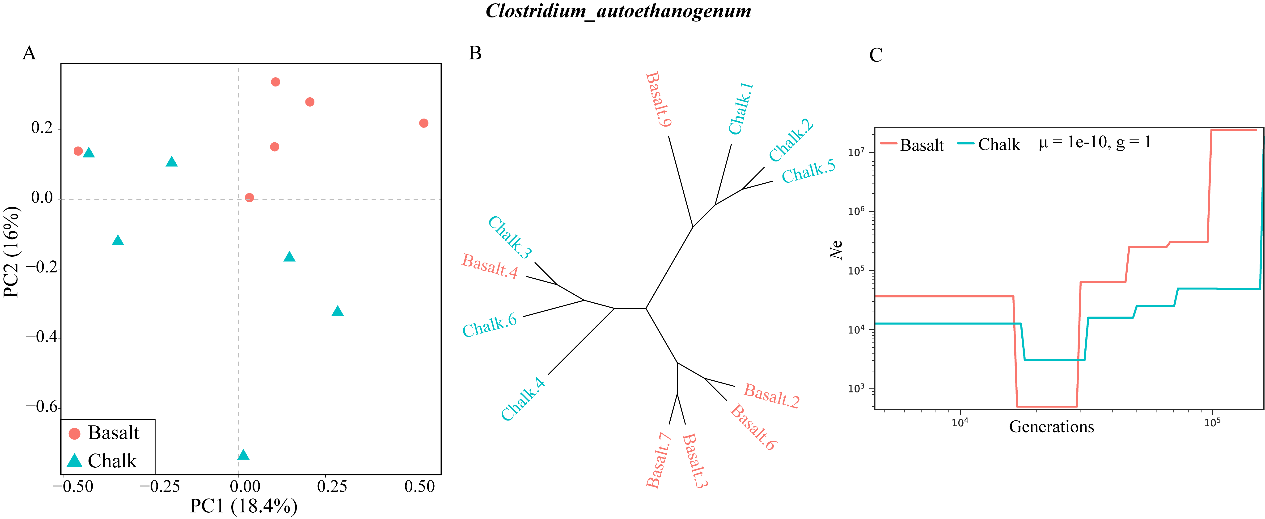


**Supplementary Figure 27.** Sympatric divergence of the single bacteria of *Clostridium autoethanogenum*. (A) Principal component analysis shows samples from basalt clustered together and samples from chalk were in one cluster. (B) Phylogenetic tree of the chalk and abutting basalt populations. (C) Population demographic history.


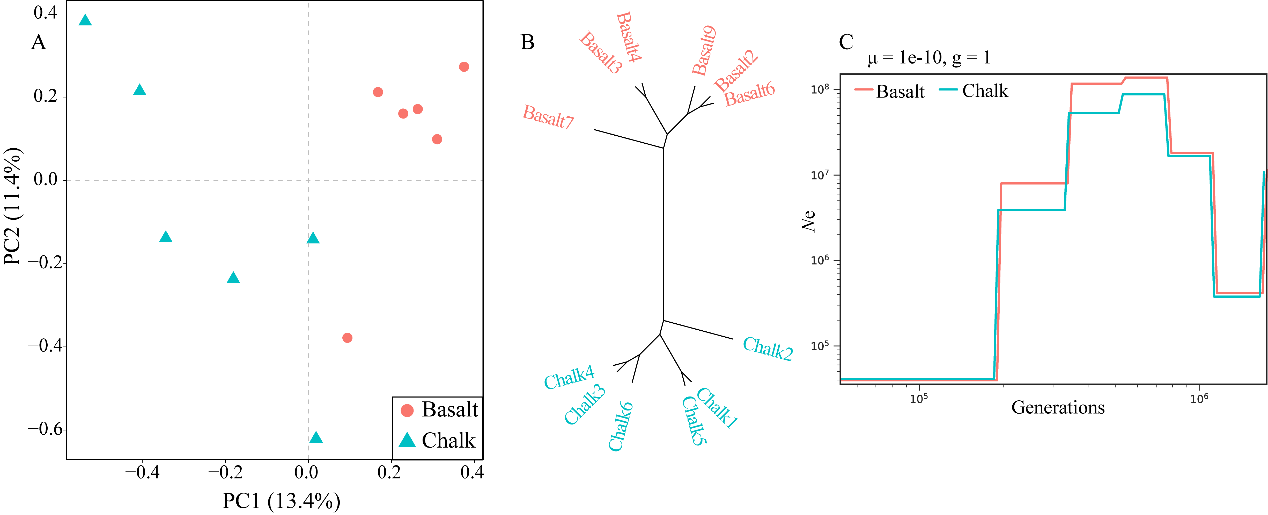


**Supplementary Figure 28.** Sympatric divergence of the single bacteria of *Clostridium innocuum*. (A) Principal component analysis shows samples from basalt clustered together and samples from chalk were in one cluster. (B) Phylogenetic tree of the chalk and abutting basalt populations. (C) Population demographic history.


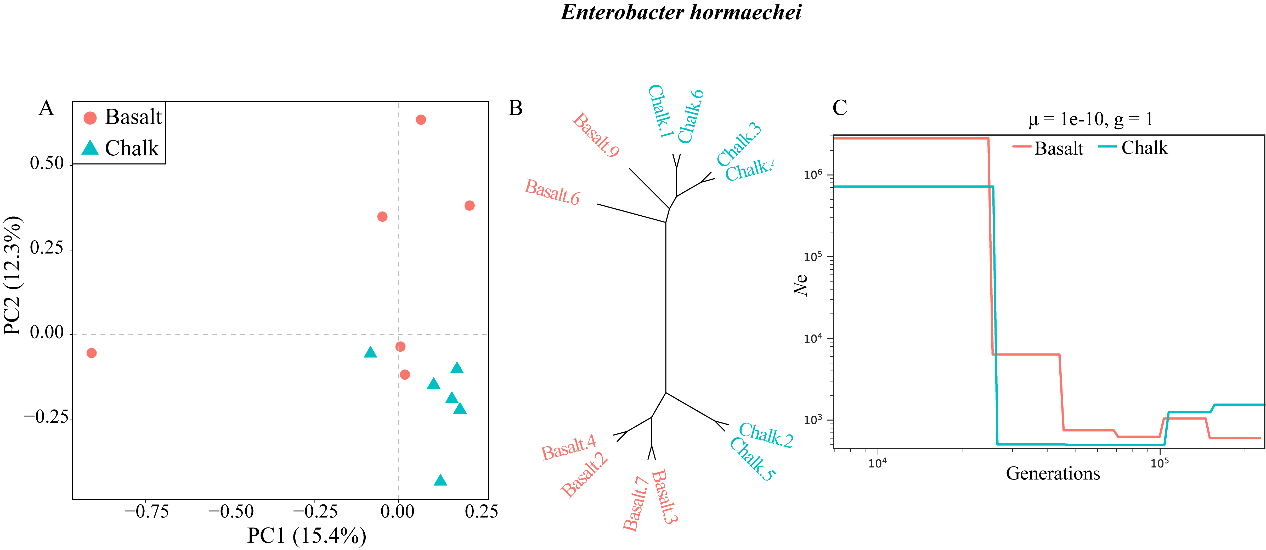


**Supplementary Figure 29.** Sympatric divergence of the single bacteria of *Enterobacter hormaechei*. (A) Principal component analysis shows samples from basalt clustered together and samples from chalk were in one cluster. (B) Phylogenetic tree of the chalk and abutting basalt populations. (C) Population demographic history.


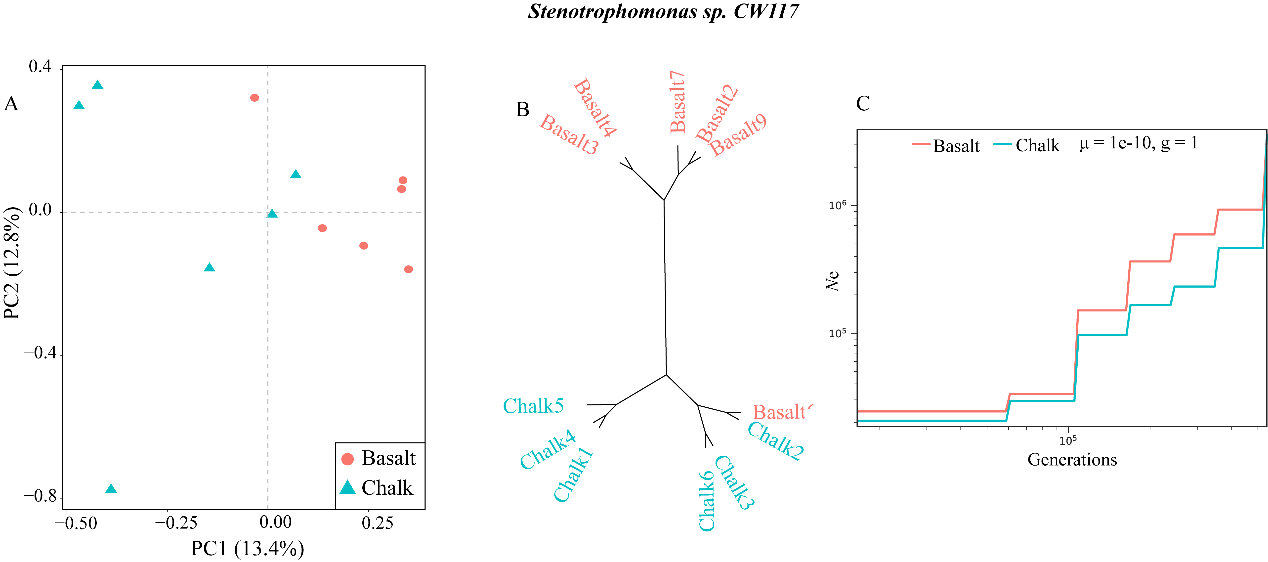


**Supplementary Figure 30.** Sympatric divergence of the single bacteria of *Stenotrophomonas* sp. CW117. (A) Principal component analysis shows samples from basalt clustered together and samples from chalk were in one cluster. (B) Phylogenetic tree of the chalk and abutting basalt populations. (C) Population demographic history.


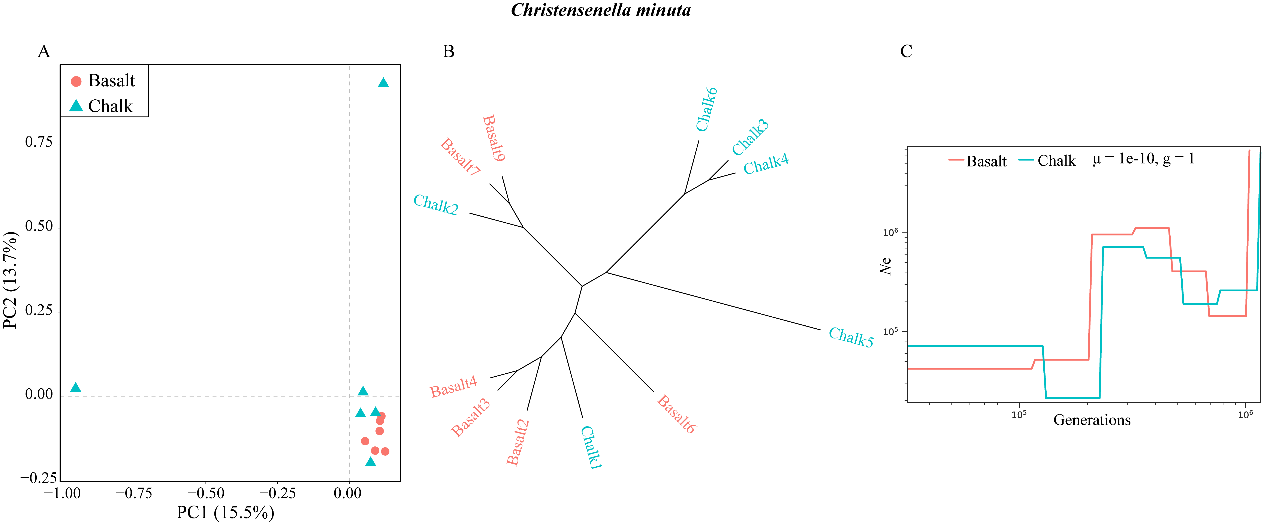


**Supplementary Figure 31.** Sympatric divergence of the single bacteria of *Christensenella minuta*. (A) Principal component analysis shows samples from basalt clustered together and samples from chalk were in one cluster. (B) Phylogenetic tree of the chalk and abutting basalt populations. (C) Population demographic history.


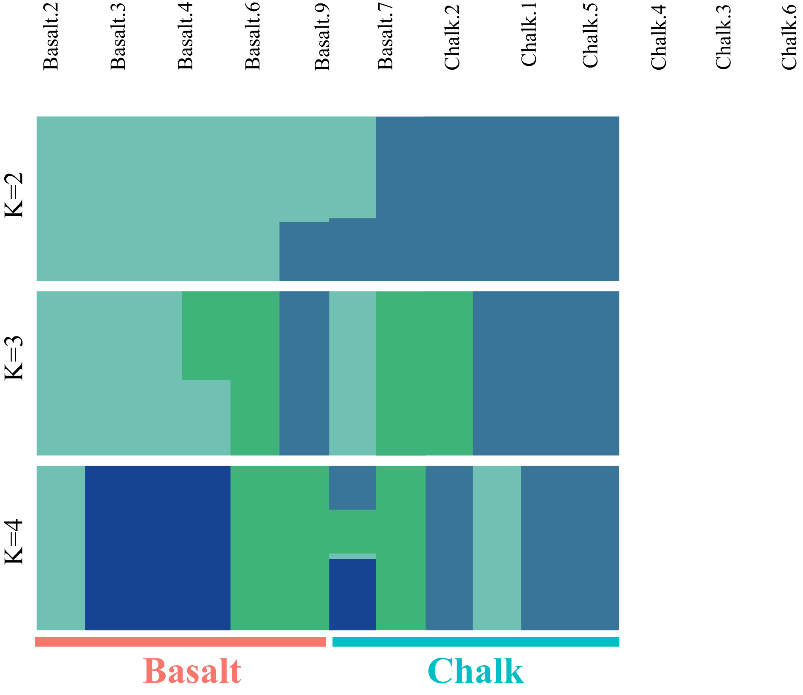


**Supplementary Figure 32.** Population genetic structure of *Flavonifractor plautii* in gut.


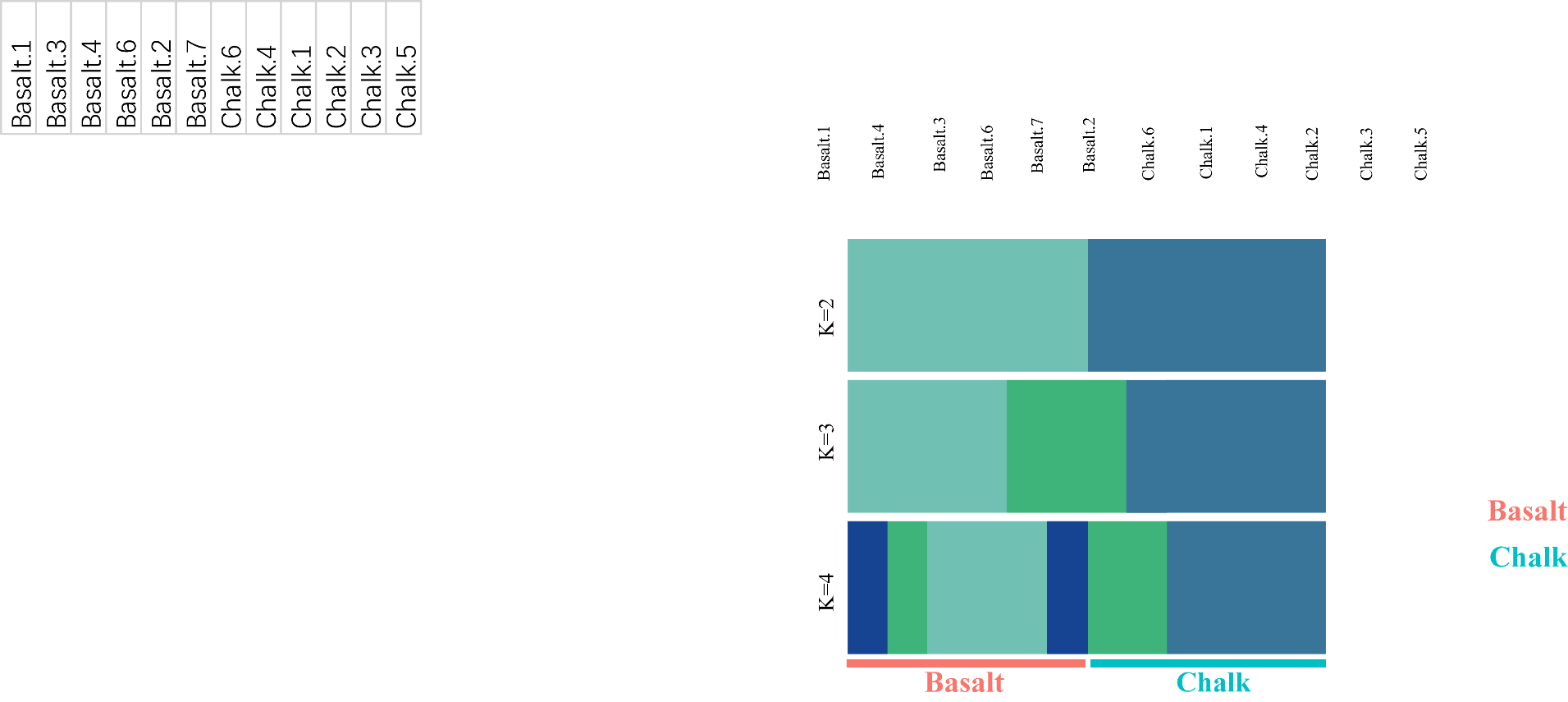


**Supplementary Figure 33.** Population genetic structure of *Flavonifractor plautii* in soil.


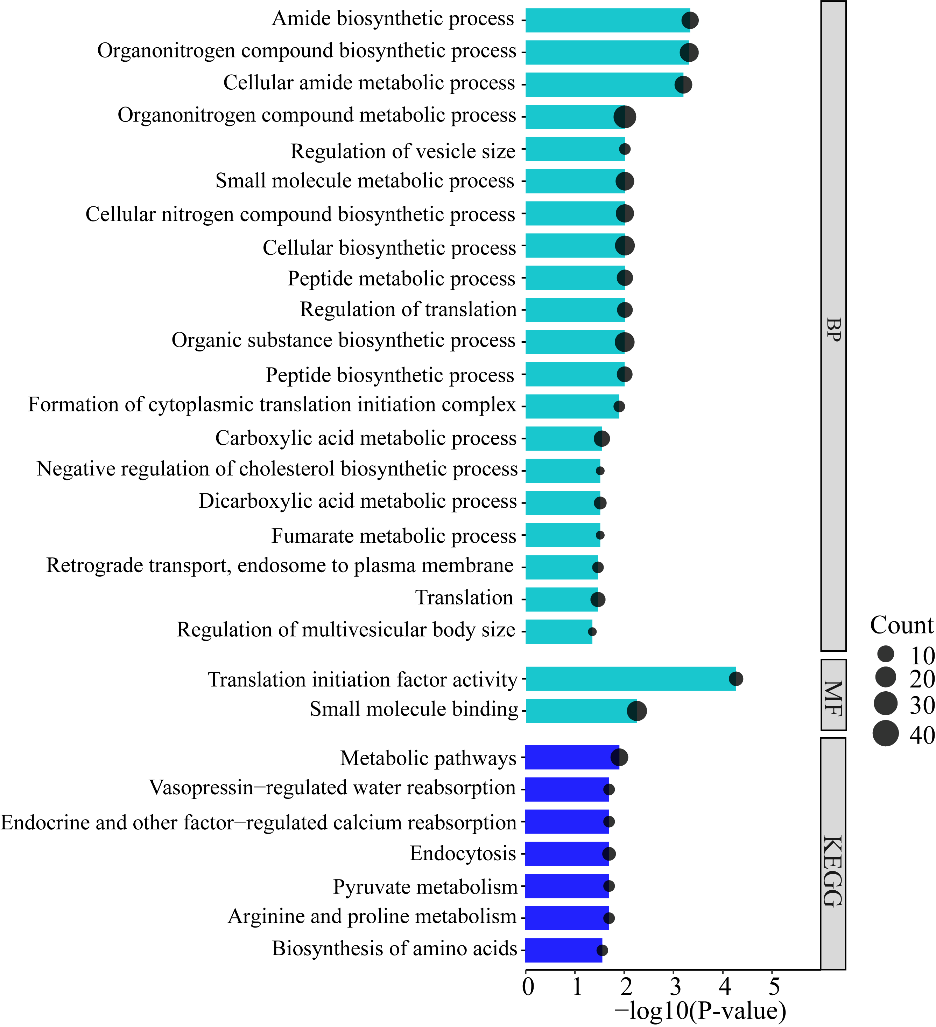


**Supplementary Figure 34.** The functional enrichment of the differential proteins, from top to bottom, is the Biological Process, Cellular Components of Gene Ontology (GO) and KEGG pathways.


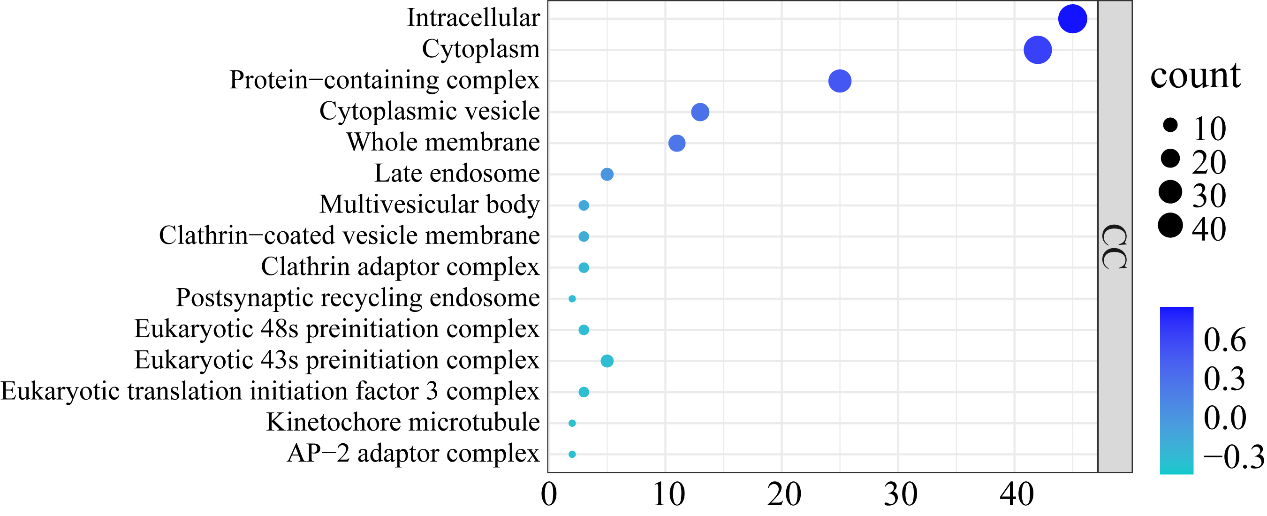


**Supplementary Figure 35.** Cellular Components of GO enrichment analysis of the significant differential proteins.

**Supplementary Table 1.** Statistics of raw sequenced reads

| Sample | Raw Reads | Clean Reads | Clean/Raw (%) | Min Length | Max Length | Mean Length |
| --- | --- | --- | --- | --- | --- | --- |
| Basalt-2 | 48,985,080 | 44,714,902 | 91.28 | 150 | 150 | 150 |
| Basalt-3 | 48,983,976 | 44,786,176 | 91.43 | 150 | 150 | 150 |
| Basalt-4 | 48,985,346 | 44,494,620 | 90.83 | 150 | 150 | 150 |
| Basalt-6 | 50,621,714 | 44,491,850 | 87.89 | 150 | 150 | 150 |
| Basalt-7 | 57,153,406 | 43,870,744 | 76.76 | 150 | 150 | 150 |
| Basalt-9 | 57,152,504 | 43,572,784 | 76.24 | 150 | 150 | 150 |
| Chalk-1 | 57,152,566 | 43,830,024 | 76.69 | 150 | 150 | 150 |
| Chalk-2 | 58,785,728 | 44,630,000 | 75.92 | 150 | 150 | 150 |
| Chalk-3 | 50,615,294 | 43,810,784 | 86.56 | 150 | 150 | 150 |
| Chalk-4 | 52,248,540 | 44,837,874 | 85.82 | 150 | 150 | 150 |
| Chalk-5 | 52,248,346 | 45,060,196 | 86.24 | 150 | 150 | 150 |
| Chalk-6 | 52,248,778 | 45,108,356 | 86.33 | 150 | 150 | 150 |

**Supplementary Table 2.** Statistics of assembled contigs

| Samples | No. of contigs | Total assembly length (bp) | Average contigs size (bp) | Contig N50 | Max contig size (bp) | Min contig size (bp) |
| --- | --- | --- | --- | --- | --- | --- |
| Basalt-2 | 714,876 | 583,495,032 | 816 | 932 | 239,425 | 200 |
| Basalt-3 | 662,769 | 617,248,762 | 931 | 1,200 | 343,274 | 200 |
| Basalt-4 | 600,090 | 578,366,279 | 964 | 1,294 | 356,061 | 200 |
| Basalt-6 | 628,901 | 597,566,275 | 950 | 1,269 | 405,596 | 200 |
| Basalt-7 | 642,131 | 589,040,727 | 917 | 1,192 | 225,361 | 200 |
| Basalt-9 | 628,250 | 568,828,541 | 905 | 1,150 | 350,032 | 200 |
| Chalk-1 | 572,527 | 517,603,755 | 904 | 1,128 | 247,903 | 200 |
| Chalk-2 | 557,558 | 541,228,252 | 971 | 1,318 | 444,376 | 200 |
| Chalk-3 | 471,418 | 500,804,361 | 1,062 | 1,704 | 536,665 | 200 |
| Chalk-4 | 437,020 | 497,040,511 | 1,137 | 1,887 | 451,008 | 200 |
| Chalk-5 | 485,102 | 524,799,589 | 1,082 | 1,766 | 489,193 | 200 |
| Chalk-6 | 450,216 | 510,586,351 | 1,134 | 2,017 | 310,981 | 200 |
